# Unraveling the Marine Microplastic Cycle: The First Simultaneous Data Set for Air, Sea Surface Microlayer, and Underlying Water

**DOI:** 10.1021/acs.est.3c05002

**Published:** 2023-10-18

**Authors:** Isabel Goßmann, Karin Mattsson, Martin Hassellöv, Claudio Crazzolara, Andreas Held, Tiera-Brandy Robinson, Oliver Wurl, Barbara M. Scholz-Böttcher

**Affiliations:** † Institute for Chemistry and Biology of the Marine Environment (ICBM), Carl von Ossietzky University of Oldenburg, P.O. Box 2503, Oldenburg 26111, Germany; ‡ Department of Marine Sciences, University of Gothenburg, Kristineberg 566, Fiskebäckskil 45178, Sweden; § Chair of Environmental Chemistry and Air Research, Technische Universität Berlin, Berlin 10623, Germany; ∥ GEOMAR Helmholtz Center for Ocean Research Kiel, Wischhofstraße 1-3, Kiel 24148, Germany; ⊥ Center for Marine Sensors, Institute for Chemistry and Biology of the Marine Environment (ICBM), Carl von Ossietzky University of Oldenburg, Wilhelmshaven 26382, Germany

**Keywords:** microplastics, tire wear particles, sea surface microlayer, air/water interface, pyrolysis-GC/MS, mass-based quantification

## Abstract

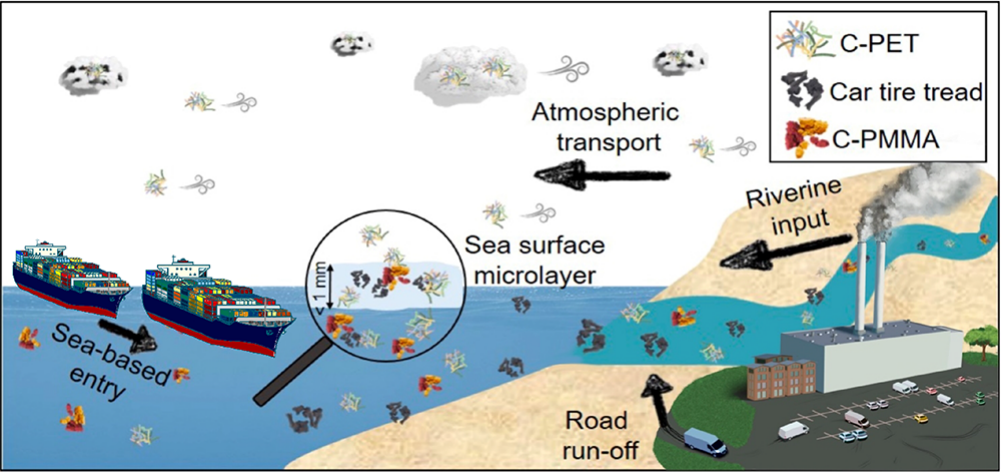

Microplastics (MP) including tire wear particles (TWP) are ubiquitous. However, their mass loads, transport, and vertical behavior in water bodies and overlying air are never studied simultaneously before. Particularly, the sea surface microlayer (SML), a ubiquitous, predominantly organic, and gelatinous film (<1 mm), is interesting since it may favor MP enrichment. In this study, a remote-controlled research catamaran simultaneously sampled air, SML, and underlying water (ULW) in Swedish fjords of variable anthropogenic impacts (urban, industrial, and rural) to fill these knowledge gaps in the marine-atmospheric MP cycle. Polymer clusters and TWP were identified and quantified with pyrolysis-gas chromatography–mass spectrometry. Air samples contained clusters of polyethylene terephthalate, polycarbonate, and polystyrene (max 50 ng MP m^–3^). In water samples (max. 10.8 μg MP L^–1^), mainly TWP and clusters of poly(methyl methacrylate) and polyethylene terephthalate occurred. Here, TWP prevailed in the SML, while the poly(methyl methacrylate) cluster dominated the ULW. However, no general MP enrichment was observed in the SML. Elevated anthropogenic influences in urban and industrial compared to the rural fjord areas were reflected by enhanced MP levels in these areas. Vertical MP movement behavior and distribution were not only linked to polymer characteristics but also to polymer sources and environmental conditions.

## Abstract 2

Microplastics including tire wear particles were found in all studied compartments and seemed to distribute vertically based on their sources, environmental conditions, and marginally by their properties.

## 1 Introduction

Microplastics (MP) include synthetic particles, fragments, and fibers with a diameter between 1 μm and 5 mm originating from highly diverse polymer applications.^1^ A small group of polymers covers more than 80% of the plastic demand in Europe.^2^ This group contains well-studied thermoplastics, including polyethylene (PE), polypropylene (PP), polystyrene (PS), poly(ethylene terephthalate) (PET), poly(vinyl chloride) (PVC), polycarbonate (PC), poly(methyl methacrylate) (PMMA), and polyamide (PA6). Furthermore, polyurethanes (PUR) belong to the group of high-demand polymers, which are also observed repeatedly in the environment.^3−5^ Tire wear particles (TWP) are emitted from the rubber-based tire tread through braking and acceleration processes and might form heteroaggregates with road materials (TRWP; tire and road wear particles).^1,5−9^ Both paint flakes and TWP have been added to the definition of MP.^1,10,11^

Little is known about concentrations, transport, and impact of MP in the sea surface microlayer (SML), overlying air, and underlying water (ULW). Especially when it comes to the marine MP cycle and the transport of atmospheric MP into the marine environment.^12,13^ Until now, only two mass-based studies^14,15^ and seven particle number-based studies dealing with marine atmospheric MP have been published.^12,16−22^ Mass loads up to 38 ng MP m^–3^^14^ and particle numbers in the range of 0.01 to 85 MP particles m^–3^^12,16−22^ were reported. More details are summarized in the Supporting Information (SI, Table S1).

The SML, the layer between the atmosphere and the ocean, plays a crucial role in understanding the marine-atmospheric MP cycle. It is a natural and ubiquitous organic film with a thickness of up to 1000 μm. The SML has a large impact on the physical, chemical, and biological processes of the global climate and ecosystem.^23^ Complex structures of polysaccharides, proteins, and lipids accumulate in the SML leading to gelatinous properties and also turning the SML into an interesting habitat for a variety of organisms.^23−25^

Studies concerning the accumulation of MP in the SML compared with ULW and quantitative data are scarce. Even though it is assumed that MP are enriched in the SML due to its characteristics,^23,26−29^ data for MP in the SML are so far exclusively based on particle numbers and are summarized in the SI (Table S2). Five of the six existing studies analyzed the SML without comparing it directly to underlying water or sediments. They documented among others PE and PS;^30^ paint particles characterized by alkyds and poly(acrylate/styrene);^27,31^ fibers^29^ and MP of different shapes; and fibers, fragments, and foams^32^ in the SML. Anderson et al.^28^ compared SML and ULW in estuary systems and described an enrichment of fibers in the SML.

The sampling area of this study was located on the Swedish west coast. Here, the coast lines are the most heavily impacted by marine litter in the entire northeast Atlantic.^33^The westerly facing zones showed higher concentrations of MP compared to surrounding, urban influenced coasts with less exposure to the North Sea like urban area of Uddevalla.^34^ Recent studies already emphasized the occurrence, fate, and transport of MP in these western fjord systems for water, beaches, and sediment samples.^34−39^ The general presence of MP in this particular environment made it ideal for the presented investigation.

Mass-specific MP analysis was conducted with pyrolysis-gas chromatography–mass spectrometry (Py-GC/MS) together with thermochemolysis.^3,5,9,40^ It allows simultaneous trace identification and quantification of representative MP including TWP. Additionally, copolymers and polymer applications, e.g., as binders or sealing materials, are enclosed in this thermoanalytical method. Therefore, results are given as polymer clusters related to basic key polymers, indicated with the prefix “C” as already introduced in previous publications.^41,42^ Additional information for clarification of the cluster aspect and the included polymer types is provided in the SI (Figure S3 and Table S7).

This study aims to gain deeper insights into the behavior of MP in coastal waters based on air, SML, and ULW samples. A potential enrichment of MP in SML was investigated. Simultaneous sampling of SML and ULW enabled the calculation and discussion of the enrichment factors of the individual polymers. The three different sampling areas (urban, industrial, and rural) in the western Swedish fjord systems were selected to enable a comparison of MP occurrence in relation to anthropogenic factors.

## 2 Materials and Methods

### 2.1 Sampling Area

**Figure 1:**
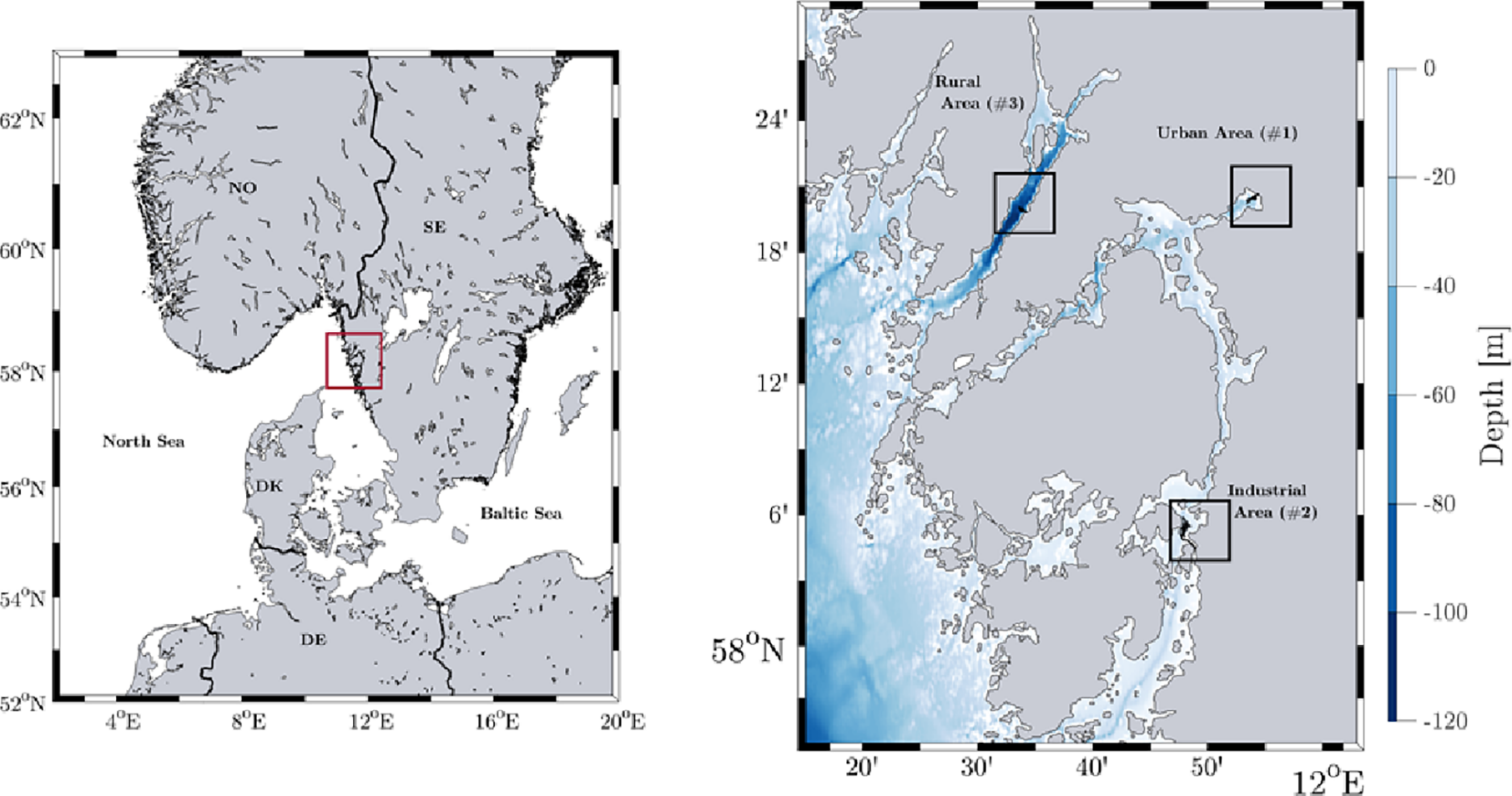
Sampling area labeled in red on the northern European map (left) and of the three fjord systems with bathymetry in meters (right). Black rectangles represent the respective sampling areas: Uddevalla Byfjord (fjord #1, urban), Askeröfjorden (fjord #2, industrial), and Gullmar Fjord (fjord #3, rural).

The sampling area of this study includes three fjord systems along the Swedish west coast north of Gothenburg (Figure 1). The fjord systems differ in terms of their urban and industrial influences. The Uddevalla Byfjord (fjord #1) is a semienclosed fjord outside Uddevalla city and is entirely protected from the Skagerrak by numerous small and large islands in the archipelago. The second fjord, the Askeröfjorden (fjord #2) is located near the city of Stenungsund and hosts the largest petrochemical and plastic production industry in Sweden. It is in a similar surrounding of protecting islands, with a flow through a fjord basin. The Gullmar fjord (fjord #3) is a pristine area with rural surroundings and comparably limited local anthropogenic influence and impact. It is a bay of the Skagerrak where semibuoyant particles are known to enter and prevail in the fjord^43^ (Figure 1).

### 2.2 Sea Surface Scanner (S^3^)

SML and ULW samples were taken with the sea surface scanner (S^3^).^44^ The S^3^ is an electric-powered and remotely controlled catamaran equipped with sampling gear to collect high-volume samples from the SML and ULW. Continuously rotating glass discs are installed between the hulls of the catamaran and partially immersed in the water. Due to surface tension, the SML adheres to the glass discs, which is then removed by polycarbonate wipers. The SML was collected at a rate of 20 L per hour. ULW was sampled simultaneously at 1 m depth. Both sample streams are pumped through the same tubing system without any time difference between both streams. A detailed description of the S^3^ can be found in Ribas–Ribas et al.^44^

A single-stage impactor (custom-made, TU Berlin) connected to a compact centrifugal fan (RV45, ebm-Papst, St. Georgen, Germany) was attached to the mast of the S^3^ for the active sampling of airborne particles one and a half meters above the sea surface (SI, Figure S1). Particles were collected on an impaction substrate made of borosilicate glass (Ø 30 mm), which was coated with Apiezon-L (M&I Materials, UK) to reduce particle bounce-off. With the applied volume flow rate of 50 L min^–1^, the nominal 50% cutoff diameter of the impactor was *D*_50_ = 2.8 μm, i.e., particles of an aerodynamic diameter of 2.8 μm were collected with an efficiency of 50%. Larger particles were collected with higher efficiencies.

#### 2.2.1 Air Samples

Per day, one air sample was taken. The sampling duration was determined by the period for SML and ULW sampling and varied from 3 to 5.5 h. To mimic any secondary contamination while sampling, field blanks for the air sampling procedure were taken every day. For this purpose, a borosilicate substrate was placed in the impactor. Subsequently, the pump was switched on and immediately turned off again. All field blanks were treated as individual samples, and a field blank subtraction was performed for each day. More air sampling details are documented in the SI (Table S3).

#### 2.2.2 Water Samples

Each fjord was sampled on two consecutive days with the S^3^. The tracks of the S^3^ for each sampling day are shown in the SI (Figure S2). Due to bad weather conditions on the first of October, the S^3^ was operated while tied to the quay. Each day, three samples (I–III) of SML and ULW water were taken. The S^3^ pumped the water samples into prerinsed PE-canisters with a 10 L volume. For preservation, the samples were poisoned with copper sulfate (CuSO_4_·5 H_2_O, 1.5 mg L^–1^, Sigma-Aldrich, Germany). Detailed information concerning the SML and ULW sampling is presented in the SI (Table S4). Blanks of the S^3^ were taken by pumping prefiltrated water (0.3 μm glass fiber filter, Whatman, Altmann Analytikal, Germany; pretreated in a muffle furnace at 500 °C for 4 h) through the flow-through system of the S^3^. More information about the blank procedure and reported secondary contamination is displayed in the SI (Text Section S1, Table S10).

Water samples of each sampling day included three SML and three ULW samples of 10 L each, taken in consecutive sets (SI, Table S4). Due to complex currents, heterogeneous particle distribution, and the necessary time for sampling, the samples did not fulfill the requirements for triplicates. Sample sets could not be collected in the exact same areas and hence represented slightly different water masses (see SI, Figure S2, GPS data). Accordingly, respective samples of SML and ULW were not treated in triplicate but were combined and considered as one sample.

### 2.3 Sample Treatment

#### 2.3.1 Avoidance of Secondary Contamination; Laboratory Blanks

All solutions and chemicals used for sample treatment were always freshly prepared and prefiltered (0.3 μm) to avoid secondary contamination and ensure consistent quality. To document any possible contamination during the sample preparation process, several full procedural laboratory blanks (*n* = 11) were prepared. Laboratory gear was exclusively made of glass, stainless steel, or TEFLON and was freshly rinsed with prefiltered water and ethanol alcohol (EtOH, 96%; university of Oldenburg laboratory supplies, Germany) before usage. During the preparation, all used beakers and filtration units were consistently covered with aluminum foil. Cotton laboratory coats were worn at all times, and synthetic fibers were prohibited in the laboratory.

#### 2.3.2 Air Samples

Air samples were removed from the borosilicate substrate by mechanically wiping off the sample with glass fiber filters (6 mm diameter, 1 μm pore size, Pall Life Sciences, VWR International, Germany; pretreated in a muffle furnace at 500 °C for 4 h), which were soaked with various prefiltrated solvents (petroleum ether, Carl Roth GmbH + Co. KG, Germany; dichloromethane, Sigma-Aldrich, Germany). All glass fiber filter-wiping pads were combined and transferred to a stainless steel pyrolysis cup.

#### 2.3.3 Water Samples

Samples were filtered on stainless steel filters (pore size 10 μm, Ø 4.7 cm; Rolf Körner GmbH, Germany) and subsequently treated with 20 mL of hydrogen peroxide (30% (v/v); university of Oldenburg laboratory supplies, Germany) and 20 mL of hydrochloric acid (2 M, VWR International, Germany). The filter residues were transferred onto a glass fiber filter (15 mm diameter, 1 μm pore size, Pall Life Sciences, VWR International, Germany; pretreated in a muffle furnace at 500 °C for 4 h) under thorough rinsing with EtOH and finally rinsed with 5 mL of petroleum ether. The glass fiber filter together with the resulting filter cake was folded and placed in a stainless steel pyrolysis cup (Eco Cups 80 LF, Frontier Laboratories, Japan).

### 2.4 Polymer Identification, Quantification, and Calibration with Py-GC/MS

Measurements were conducted with Py-GC/MS according to established methods,^9,40^ and details are given in the SI (Table S6). For internal process standardization, 20 μL of deuterated polystyrene solution (dPS, 125 μg mL^–1^ in dichloromethane) was added directly into each pyrolysis cup. Furthermore, a thermal online-transesterification (methylation) was performed by adding 20 μL of tetramethylammonium hydroxide solution (TMAH, 12.5% in methanol (MeOH); Sigma-Aldrich, Germany) for particular improvement of detection sensitivity for PET and PC.^3^

Polymer clusters of PE, PP, PS, PET, PMMA, PC, and PA6 were identified and quantified as described^3,11,40,45^ and indicated with the prefix “C-". PURs were represented by C-MDI-PUR, which combines all aromatic PURs based on methylene diphenyl diisocyanate (MDI) as isocyanate moiety.^45^ Identification and quantification of car and truck tire tread were conducted as described previously^5,9^ and were thereafter presented in the results as TWP representing a sum of both tire types. Calibration was based on both particulate and dissolved standards. Further information concerning identification, plastic standards used for quantification, calibration, and limits of detection and quantification are provided in the SI (Text Section S2, Tables S5 and S7–S9).

The C-PVC indicator ion naphthalene, known to be rather nonspecific, is also a suspected pyrolysis product of distinct polymeric soot residues, which are universally present in the environment. Its restrictions have already been shown in previous studies, highlighting that refractory polymeric material of certain carbon blacks, e.g., chimney soot, are capable of releasing naphthalene during pyrolysis.^5^ A clear quantification of C-PVC is impossible until a convincing correction factor is established for near-shore soot sources, as overquantification might occur. Accordingly, C-PVC was excluded from the results of this study and subsequent discussion. The borosilicate substrates of the air sampler were covered with Apiezon-L, a high-boiling lubricating grease on a hydrocarbon basis. Its dominating signals overlaid the homologue series of C-PE indicators. Therefore, the quantification of C-PE was omitted for the air samples. However, respective peak areas, indicative for C-PE in both field blanks and air samples, appeared in similar orders of magnitude and pointed to very low, if at all present, C-PE contents.

## 3 Results and Discussion

### 3.1 MP Concentration in Air Samples

**Figure 2:**
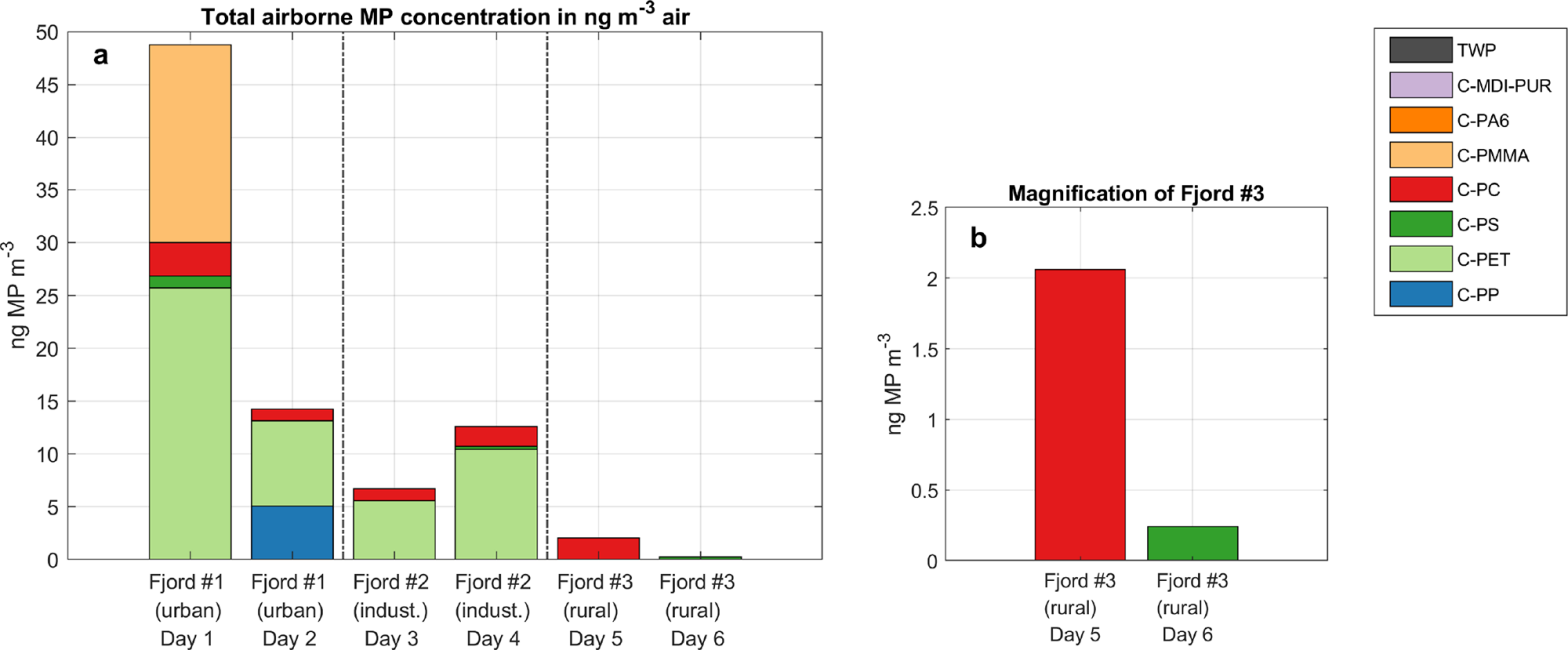
(a) Total airborne MP concentration in ng m^–3^ in the three different fjord systems; (b) magnification of fjord #3 (no quantification of C-PE, c.f. 2.5).

Atmospheric deposition contributes to the MP particle burden in the ocean, and it is strongly dependent on the particle size and concentration in the air. Active impactor sampling and subsequent Py-GC/MS yield information about the total MP mass concentration in the air. The active sampled particles from the ambient air of the three Swedish fjords showed detectable MP loads on each sampling day (Figure 2; SI, Table S11).

The urbanized fjord #1 stood out with the highest total mass loads, particularly with 49 ng MP m^–3^ on the first sampling day. In fjord no. 2 (industrial), the MP concentration ranged from 7 to 13 ng MP m^–3^. The lowest MP mass loads were found in fjord #3 (rural) with concentrations below 5 ng of MP m^–3^. Compared to measured total particulate mass concentrations of 11,800 ng m^–3^ (PM10) determined in the air in Råö, south of Gothenburg,^46^ the observed mean MP mass concentrations in the air samples contributed 0.3 (fjord #1) and 0.01% (fjord #3) to the total atmospheric particle load. To estimate the atmospheric MP deposition flux, we multiplied the observed MP concentrations and the particle deposition velocity were multiplied. Assuming a particle deposition velocity of 0.003 m s^–1^, approximated for an aerodynamic diameter of *D* = 10 μm according to Hinds,^47^ the atmospheric MP deposition flux could be very roughly estimated to range from below 0.015 ng m^–2^ s^–1^ in fjord #3 to 0.15 ng m^–2^ s^–1^ in fjord #1.

The most prominent polymer clusters detected in the air samples were C-PET and C-PC. In fjords #1 and #2 (urban and industrial), C-PET dominated with 69%. Additionally, C-PS was frequently detected. C-PMMA and C-PP were identified in fjord #1, the urban area. Other polymer clusters including C-PA6, C-MDI-PUR, and TWP were completely absent in all air samples.

Potential sources for C-PET in the (marine) atmosphere are fibers from, e.g., textiles or ropes. These were already described to occur in high abundances in the air,^5,14,22,48,49^ to travel via aeolian transport, and finally, to deposit on the ocean’s surface.^13,50^ A visual check of the borosilicate substrates revealed the presence of fibers, which might be directly related to the C-PET concentration in the samples (SI, Figure S4). PC is often used in the building, construction, and electronic sector.^2^ These rather long-living products are unlikely responsible for the universally present C-PC concentration in air samples. The observed contamination is rather related to abrasion from epoxide-based coatings, characterized by bisphenol A (BPA), the same building polymer backbone as for pure PC.^11^ A direct impact of molecular BPA, detected in environmental samples,^51^ could not be evaded for the air samples of this study. During regular sample processing, intensive washing steps remove low molecular organic materials including BPA. Here, air samples were directly transferred into pyrolysis cups, and no further treatment was applied.

A comparison with literature data is challenging as various sampling and analytical methods are used for the comparatively few studies available on MP pollution in the atmosphere. The two mass-based MP study by Goßmann et al.^14^ and Caracci et al.^15^ analyzed MP in Atlantic air and described concentrations up to 37.5 and 51.7 ng MP m^–3^, respectively. While the Atlantic air was noticeably polluted with TWP^14^ in some areas (37.2 and 13.7 ng TWP m^–3^) and polyisoprene^15^ (PI; 51.7 ng PI m^–3^), also a possible indicator for the presence of TWP, no indication for TWP was found in the air samples of the Swedish fjord samples. In contrast to the study by Goßmann et al.,^14^ the other detected polymer clusters were present in higher orders of magnitude in the Swedish fjords. However, the overall composition of polymer clusters detected in the air samples was similar. Especially, the ubiquity of C-PET and C-PS was demonstrated again.^14^ The predominance of C-PET was divergent form the findings by Caracci et al.^15^ Its total absence in any sample of their study was not in line with the data presented here, in Goßmann et al.,^14^ and other particle related studies (SI, Table S1). This might be attributed to the different and highly complementary analytical approaches of both studies with respect to polymer preconcentration and detection principle. The method used by Caracci et al.^15^ is based on stepwise suspect screening of polymer chain profiles via size exclusion chromatography HRMS coupling of toluene soluble plastics. Py-GC/MS data enable a sensitive detection and quantification of defined base polymer clusters based on selective, thermal indicator products that combine their mass content disregarding different chain length, or their appearances as copolymers, formulations, binders, etc. (cf. SI, Figure S3). Accordingly, even mass-based studies can be challenging to compare. Publications presenting particle-number-based MP in the marine atmosphere (SI, Table S1), which were mentioned in the introduction, are here used for qualitative comparison based on relative polymer proportions, keeping in mind that a comparison of particle numbers and masses is rather uncertain. Fjord #3 is excluded since per day only traces of one polymer cluster were detected, respectively (Figure 2). Here, the relative calculation of polymer proportions would be misleading as it would be represented by 100% C-PC or 100% C-PS. Five out of seven literature studies stated PET or polyester as the most prominent polymer with documented percentages ranging from 29 to 56%.^17−19,21,22^ This coincided with the data from this study, in which C-PET was dominant. Relative proportions represented on average 69% of the polymers found. The second most dominant polymer cluster in the Swedish air samples was C-PC (Ø 11%), which matched only partly with the literature data. Only Ferrero et al.^18^ documented PC as the second most frequent polymer with around 12%. C-PS appeared in almost all air samples analyzed here, with an average of 1%. Two studies documented the PS appearance in their atmospheric samples. While Trainic et al.^20^ described PS as the main polymer found in their samples, Liu et al.^19^ mentioned PS with a percentage of 6%. Here, C-PP and C-PMMA were detected only once in one air sample. Deviating from the observations of this study, PP was determined more frequently in four out of seven studies.^17,19−21^ PMMA was described in one publication only with an average of 14%.^22^

### 3.2 MP Concentration in SML and ULW Samples

**Figure 3:**
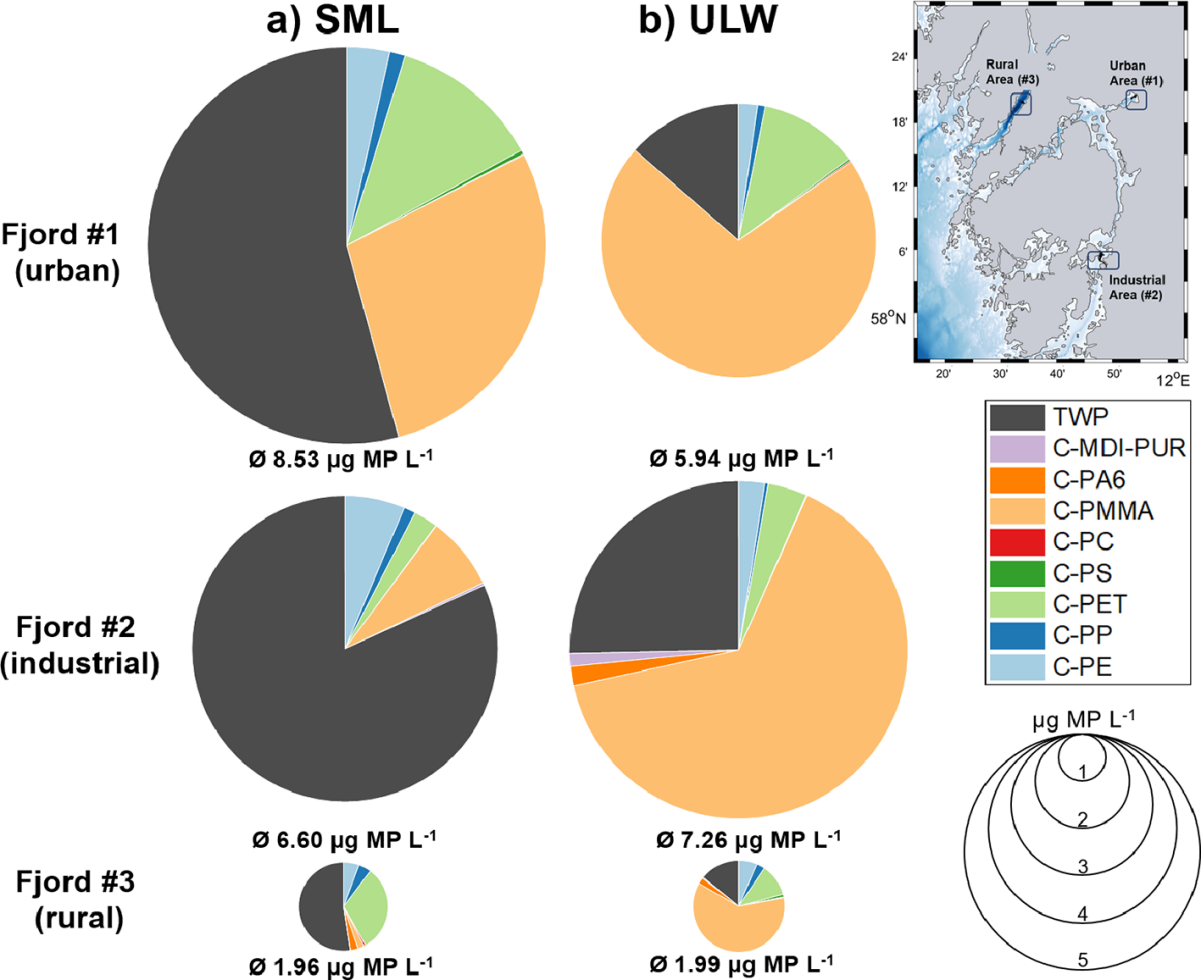
Total MP composition in (a) SML and (b) ULW for fjord #1 (urban), fjord #2 (industrial), and fjord #3 (rural) in μg MP L^–1^. The diameter of the pie charts is proportional to the total MP concentration.

MP was detected in all SML and ULW samples of the three investigated areas. All samples represented a broad variety of polymer clusters, irrespective of whether they were from the SML or the ULW. The most dominant polymers were TWP, C-PMMA, and C-PET. Polymers such as C-PE, C-PP, and C-PC were detectable in considerably lower concentrations but were universally present as well. C-PS, C-PA6, and C-MDI-PUR appeared occasionally. Total MP concentrations in the samples, regardless of SML or ULW ranged from 0.6 to 10.8 μg MP L^–1^ (Figure 3). Quantitative results of the water samples are given in the SI (Table S12).

Comparisons of observed relative TWP/TRWP proportions with modeling-based TWP/TRWP estimates, derived from the literature and specified for this environment, fitted depending on the analysis. Three different studies predicted emitted tire wear proportions (TWP and TRWP) of total emitted MP loads in the environment (both aquatic and terrestrial) for around 45%.^6,7,52^ These calculations were mainly based on global, annual production data and matched the TWP proportions of around 40% in this study. However, since C-PVC was excluded here, a comparison of the percentages is not trivial.

Besides TWP, clusters of PMMA and PET mostly dominated the Swedish water samples. The European plastic demand by resin types in 2021 showed PE with approximately 27% as the most used polymer, followed by PP (20%). PET was listed in fourth place with 6%.^2^ However, PlasticsEurope, 2022^2^ excluded fiber related polymers as well as those used for adhesives, sealants, and coatings.^2^ Therefore, neither PMMA nor cluster-related acrylates appeared as high-demand polymers. These missing components were included by Geyer^53^ where polyester, PA, and acrylic made up 14% of the global annual plastic production in 2017. Up to now, polyester fibers were the globally most produced fiber with 60.5 million metric tons in 2021.^54^ Acrylics were most likely the main source of C-PMMA in the respective fjord environments originating from both marine and terrestrial used surface coatings, paints, varnish, and road markings, which are released through the abrasion and erosion of surface coatings.^10,11,55,56^ Once abraded, they might find their way into the water body finally leading to massive C-PMMA mass loads in marine water samples.^11,56^ C-PET mass loads were unlikely emitted by PET bottles, since Sweden has an efficient recycling system.^2^ A more plausible source were fibers used for textiles, which were emitted into the aquatic environment via short- and long-range atmospheric deposition, effluents of wastewater treatment plants, surface runoff, and the release of gray water from ships.^50,57,58^ A visual check supported the presence of fibers on the respective filter cakes and revealed their frequent occurrence (SI, Figure S5). Additionally, a recently published study about MP in the Kattegat/Skagerrak region reported a clear dominance of polyester fibers in surface waters near the here sampled fjords.^59^ The top high-demand polymers PE and PP, mostly used for packaging purposes, were constantly detected in the samples. However, their share of the total MP load is comparably low. Since Sweden banned the landfill of plastics in 2005 (<1% of plastic waste treatment),^2^ waste mismanagement is supposed to be a rather neglectable source. This is in accordance with the minor presence of these clusters in the samples. In case of fjord #2 the local polyethylene production plant might act as an additional source of C-PE contamination.^37^

Swedish surveys on micro and macro beach litter documented PE and PP as the most common polymers.^38^ Additionally, PE and PP were the most commonly identified polymers in previous microspectroscopy studies in the area, although previously limited to larger size fractions (>50 or >300 μm).^34,35,38,39^ Hence, since this study sampled with lower size limits (>10 μm) albeit with lower sampling volumes and the fact that Py-GC/MS and spectroscopy may have different and complementary detection capabilities, the difference in detected polymer clusters is not surprising in general.^41^

#### 3.2.1 Comparison of Sampling Areas

Here, the results are considered concerning different anthropogenic influences and land use of the three different sampling areas in an urban environment (fjord no. 1), an industrial site (fjord no. 2), and a rural area (fjord #3).

Total observed MP mass loads were higher in those environments strongly influenced by anthropogenic and industrial factors (Figure 3). On average, fjord #1 contained 8.53 μg MP L^–1^ SML and 5.94 μg MP L^–1^ ULW. Fjord #2 was contaminated in similar orders of magnitude (6.60 μg MP L^–1^ SML and 6.06 μg MP L^–1^ ULW). The rural fjord no. 3 with rather touristic use was less polluted with averages of 1.96 μg MP L^–1^ SML and 1.99 μg MP L^–1^ ULW. In addition, one-way analysis of variance (ANOVA) and multiple comparison tests after Tukey were performed with MATLAB to determine significant differences between the three different fjords for SML and ULW samples. For this purpose, the sum of all polymer clusters was calculated and given as total MP concentration. For each fjord, at least six subsamples were available for SML and ULW, respectively, and included in the tests (SI, Table S4). Since the samples were no true replicates, the statistical results serve only as an approximation. The SML samples showed significant differences between fjord #3 compared to fjord #1 and #2 (*p*-value = 0.004). Due to greater variabilities within the ULW in the individual fjords, no significant differences were observed between the fjords. ANOVA and multiple comparison tests are displayed in the SI (Figures S7 and S8).

The results reflect the geophysical properties and expected emissions of the sampling areas. The sampling area of Fjord #1 was located outside the commercial shipping harbor, with a busy bridge leading across the fjord. Additionally, a small creek passing through Uddevalla flows into the fjord. The creek was influenced by both traffic of the city and a yacht harbor in the creek. Road traffic led most likely to high concentrations of TWP, especially in the SML (Ø 4.62 μg L^–1^). Particularly large C-PMMA mass loads in the ULW (Ø 4.23 μg L^–1^) in fjord #1 might have emerged from antifouling paints from ships and boats and from paint, coatings, adhesives, and other chemical products and applications related to harbor and city activities. Due to the urban environment, a large input of textile fibers through domestic wastewater or atmospheric deposition most likely accounted for C-PET (Ø 1.05 μg L^–1^ SML, Ø 0.70 μg L^–1^ ULW). The effluents of the Uddevalla wastewater treatment plant enter the fjord in the mouth of the creek upstream of the sampling location. Previous studies identified fibers in the effluents and the surface water of this region.^43,59^

The industrial site, which surrounded the sampling area of fjord #2, included several polymer-manufacturing industries. This potentially led to the emission of a broader range of polymer types observed, particularly in the ULW. A polyethylene production plant in the area of fjord #2 produces pellets (3–4 mm) and finer particulates (<1 mm) with a well-documented, associated spill, and pollution problem.^37^ C-PE concentrations (Ø 0.42 μg L^–1^ SML, Ø 0.18 μg L^–1^ ULW) might be directly attributed to the polyolefin factory but were not significantly higher than in fjord #1. TWP values (SML Ø 5.40 μg L^–1^ and ULW Ø 1.84 μg L^–1^) could be related to increased traffic due to the delivery and collection of goods.

Roads and houses along the fjord and several small harbors with private boats characterized the rural area of fjord #3. In the innermost part of the fjord, a small municipality is located, and highway E6 passes by. Therefore, public traffic, packaging polymers, synthetic fibers, and coating from, for example, private boats and a few sewage outflows, might be sources of MP pollution. The MP composition and distribution were therefore similar to those described for fjord #1 but to a much lesser extent. Unlike fjords #1 and #2, the Gullmar fjord is connected to the Skagerrak. Accordingly, fjord #3 was rather affected by diffuse pollution instead of clearly identifiable point sources during the sampling period.

Regardless of the sampling location, the MP distribution patterns of SML and ULW samples were alike, with TWP dominating in the SML and C-PMMA in the ULW. In particular, the SML samples of fjords #1 and #2 showed an almost identical pattern (Figure 3).

### 3.3 SML vs ULW

**Figure 4:**
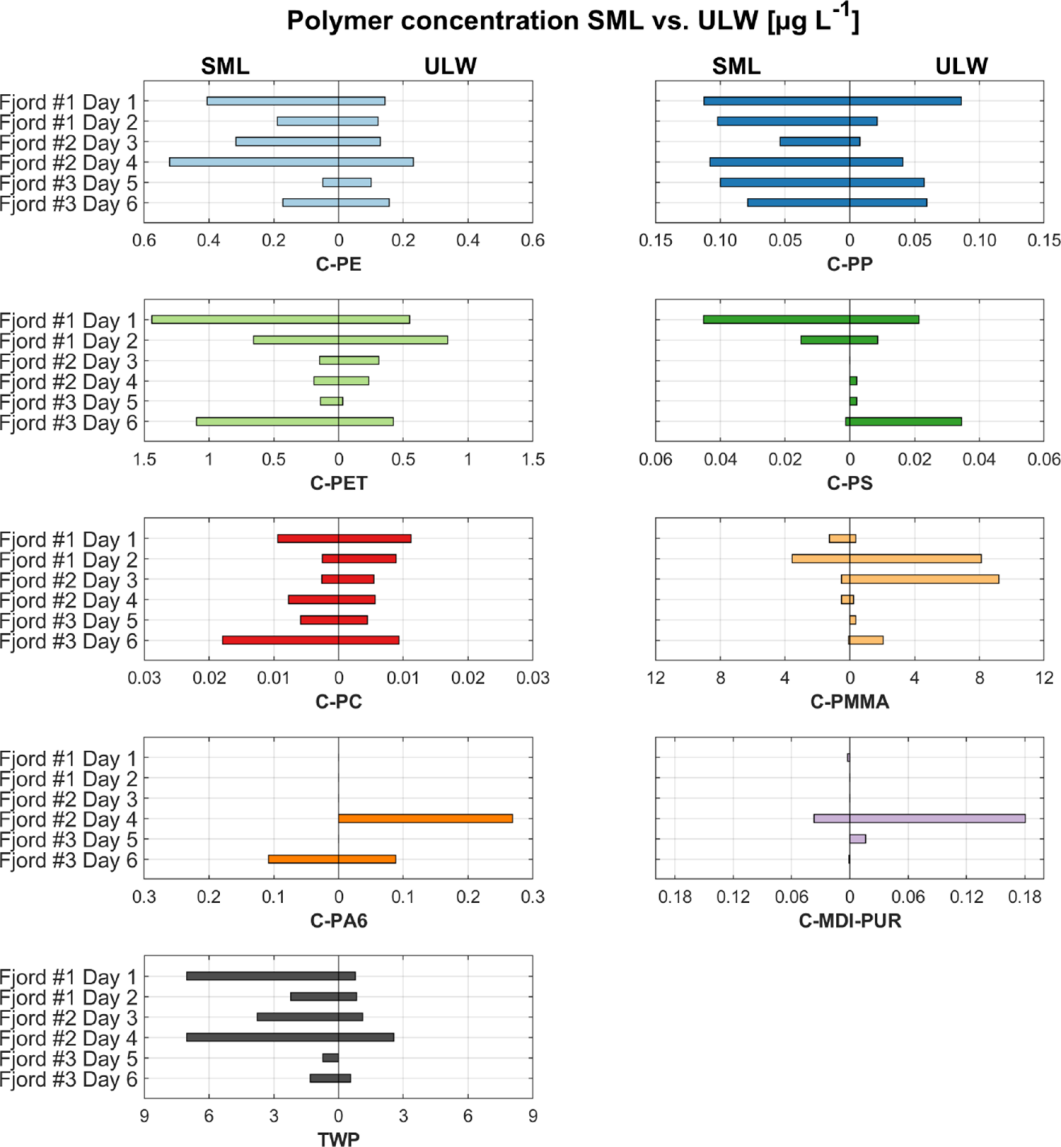
Polymer concentration in μg L^–1^. Comparison of SML and ULW for the six sampling days in fjord no. 1, fjord no. 2, and fjord #3.

To generate knowledge about the behavior of MP in the SML, polymer-specific enrichment factors (EF) were calculated with the following formula:
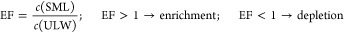


The polymer-specific EF for each sampling day is displayed in the SI (Table S13). In Figure 4, the polymer concentrations in μg L^–1^ are displayed for a direct comparison of the SML and ULW. C-PP was enriched in the SML on all sampling days with EFs between 1.3 and 7.0. C-PE and TWP were enriched in the SML in 5 out of 6 days with a max EF of 2.8 (C-PE) and 9.2 (TWP). C-PMMA and C-PS were mostly depleted in the SML (both 4 out of 6 times). C-PET and C-PC showed no clear trend, as they were equally often enriched and depleted in the SML. Insufficient data on C-PA6 and C-MDI-PUR hindered any evaluation. Total MP concentrations were not consistently higher in the SML than in the ULW, which disproves the hypothesis that MP are generally enriched in the SML. These observations also support the assumption that the density of MP does not solely affect the behavior in the water column even though some of the lighter polymers were partly enriched in the SML (e.g., PE ρ = 0.89–0.98 g cm^–3^; PP ρ = 0.83–0.92 g cm^–3^^60^). Instead, the shape and size of the respective polymer cluster have proven to be relevant. TWP are found mostly as heteroaggregates with road particles (TRWP), which have a density much higher than seawater (ρ = 1.8 g cm^–3^^61^). Still, TWP were mostly enriched in the SML. The same held true for the rather heavy polymer PET (ρ = 0.96–1.45 g cm^–3^^60^), which often occurs in the form of fibers, where the surface tension probably holds the fiber in the SML even though it is heavier. Studies on the sinking behavior of MP have confirmed lower sinking velocities when materials are in fibrous shape compared to the predicted reference for spheres, which strengthens our findings.^62,63^

### 3.4 Relative Polymer Composition in Air, SML, and ULW Samples

**Figure 5:**
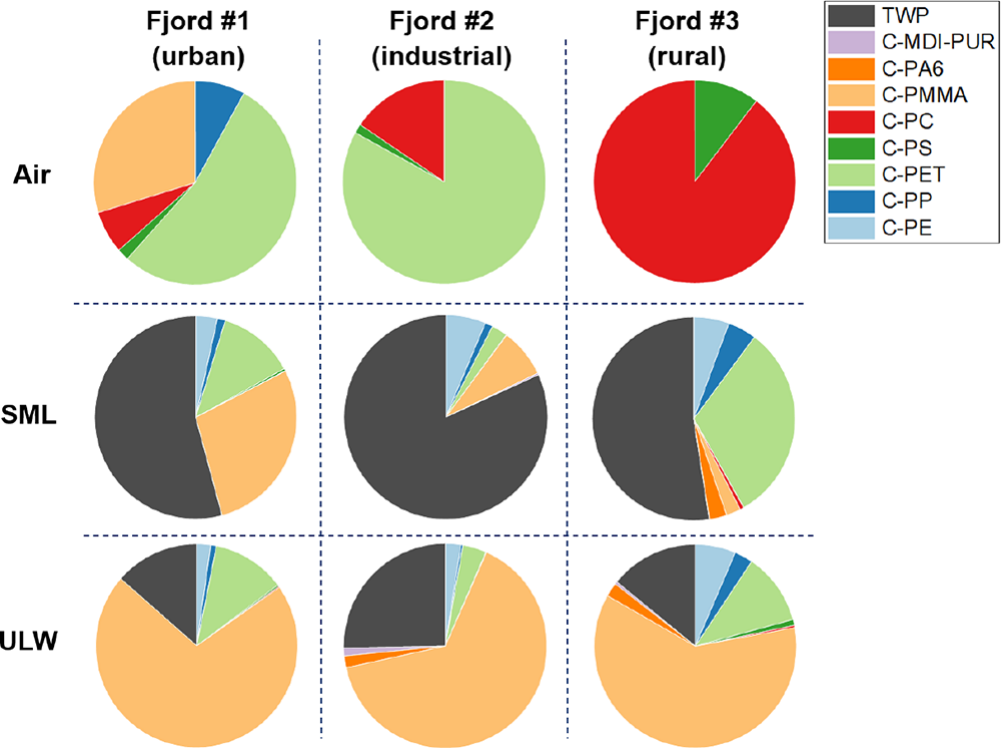
Comparison of relative MP composition in air, SML, and ULW samples in fjords 1, 2, and 3.

The MP concentrations between air and water samples differed by 2 orders of magnitude (pg L^–1^ (air, in the text given as ng m^–3^) vs μg L^–1^ (SML and ULW)). Hence, relative proportions of the sample sets air, SML, and ULW were used to get insights into the behavior of MP across the SML and the overlying air and ULW, and their vertical distribution behavior (Figure 5). In the pie charts, air, SML, and ULW sample sets taken simultaneously are arranged in three vertical columns for the respective fjords. Relative MP composition in the air, SML, and ULW samples was completely different even though sampling took place simultaneously. Air samples from fjord no. 1 and fjord no. 2 contained large proportions of C-PET, while this polymer cluster was less prominent in the related water samples. In fjord no. 3, with substantially lower absolute concentrations, the pattern differed in all three sample types. The air samples did not show any occurrences of C-PET, while the SML in fjord no. 3 had the highest relative C-PET proportions of the three fjord systems. The C-PC proportions were evident in the air samples but were rather invisible in the water samples, even though C-PC appeared ubiquitously. In all SML samples, TWP was one of the most dominant contaminants. In contrast, they were absent from all air samples. The same was applied to C-PMMA in the ULW.

### 3.5 Pathways of MP into the Marine Environment

In the following paragraph, possible pathways into the marine environment are discussed with a focus on the three most dominant occurring MP types, C-PET, C-PMMA, and TWP.

C-PET was the dominant polymer cluster present in the air samples. This supports the hypothesis that atmospheric transport may be an important entry point for C-PET to the marine environment.^50^ In the water samples, C-PET was the third most frequent polymer cluster but showed neither an enrichment nor a depletion in the SML. It is well documented that the fibers enter the studied areas in large quantities through effluents of wastewater treatment plants,^43,58^ but also, input from gray water emitted by ships is discussed in the literature.^57^ These effluents are usually discharged directly into the water body below the stratified mixed layer and may not reach the SML. Accordingly, C-PET may enter the SML through atmospheric deposition, with a certain residence time in the SML, and subsequently sink into the ULW. Alternatively, it is directly discharged into the ULW via wastewater treatment plants. These processes are partially energy-driven (wind and waves) as well as influenced by biofouling and partly affected by density, which is also described in the literature.^62,63^ Laboratory experiments investigated the residence time of small, pristine PET particles (<100 μm) in the SML showing that at least particulate PET did not stay in the SML but quickly found its way into the underlying water (SI, Text Section S3, Figures S9 and S10). The naturally occurring mixture of different PET shapes and sizes in the environment may result in rather sinking particulate and more floating fibrous PET, which then leads to the observed missing enrichment in the ULW.

C-PMMA was the predominant polymer cluster in the ULW, ubiquitously occurring in the SML and only once detected in the air (fjord #1). A plausible source for this cluster is hypothesized to be related to particles from coatings and paints, e.g., from ships and surrounding industries. Emissions arise through operational abrasion, self-polishing antifouling paints, and ongoing maintenance work, e.g., sand blasting, recoating, and continuous painting work to prevent ongoing corrosion.^10,11,55,56^ Consequently, it is frequently observed in marine samples in general^10,11,56^ and in particular in the study area of Askeröfjorden in Stenungsund (fjord #2), where boat paint particles were documented using different methods.^36^ As these multilayered and multicomponent paint flakes typically have higher densities than seawater, they are less prone to accumulate in the SML.^55^ They are either directly released underwater and might be remixed into the SML. Furthermore, the detected PMMA cluster includes a broad range of acryl-containing particles from a broad range of formulations and applications that may enter the marine environment through urban/industrial runoff.

Surprisingly, TWP were exclusively found in the water samples and prevailed in the SML. This supports the assumption that these enter the marine environment mainly through terrestrial runoff and stormwater discharges, as direct pathway from roads to the aquatic environment.^50,64,65^ Tire rubber has an approximate density of 1.2 g cm^–3^.^61^ Accordingly, an accumulation in the ULW is expected, but instead, an enrichment of TWP in the SML was observed. However, the absence of TWP in the air samples of this study does not necessarily mean that TWP is not entrained into the environment via atmospheric transport. In this study, volumes of sampled air were comparatively small (9.81–18.24 m^3^), while TWP calibrated as tire tread directly has a high detection limit concerning its analytical indicator compound.^9^ Accordingly, a higher sample volume would have allowed for a clearer conclusion about the occurrence of TWP in air. In contrast to this study, TWP occurrences in the marine atmosphere have been both modeled^66^ and experimentally proven.^14^ The latter study detected TWP particles even in northern Atlantic air samples of volumes >500 m^3^. However, the given results matched earlier statements that allocated stormwater discharge and road runoff as the predominant entry pathways of TWP in the aquatic environment, while only low quantities ended up in the aquatic environment through atmospheric deposition.^50,64,65^

The sum of observations made in this study led to the conclusion that MP particle properties (type, shape, size, and density) and also the individual input pathways are of equally great importance for the vertical distribution and transport in the respective environmental compartments. The studied SML together with the overlying air and ULW gave new insights into sources, fate, and pathways of MP through the marine environment.

## Supporting Information Available

Single file containing information on sampling locations, sample details, Py-GC/MS method parameters, polymer standards, calibration measurements, tables with quantitative data, and detailed information about field blank procedures and additional experiments concerning the residence time of PET in the SML (PDF)
