# Supplementary material for: Unraveling the Marine Microplastic Cycle: The First Simultaneous Data Set for Air, Sea Surface Microlayer, and Underlying Water
Source: Environ Sci Technol. 2023 Oct 18;57(43):16541–51. doi: 10.1021/acs.est.3c05002 (PMC10620994; doi:10.1021/acs.est.3c05002)
Supplement: Supplementary file 1 — es3c05002_si_001.pdf [file es3c05002_si_001.pdf]

Supporting Information (SI)

A contribution to unravel the marine microplastic cycle – A first coherent data set for air, sea surface microlayer, and underlying water

Isabel Goßmann[1,5], Karin Mattsson[2], Martin Hassellöv[2], Claudio Crazzolara[3], Andreas Held[3], Tiera-Brandy Robinson[4], Oliver Wurl[1,5], Barbara M. Scholz-Böttcher[1]*

[1]Institute for Chemistry and Biology of the Marine Environment (ICBM), Carl von Ossietzky University of Oldenburg, P.O. Box 2503, 26111 Oldenburg, Germany

[2]Department of Marine Sciences, University of Gothenburg, Kristineberg 566, 45178 Fiskebäckskil, Sweden

[3]Chair of Environmental Chemistry and Air Research, Technische Universität Berlin, 10623 Berlin, Germany

[4]GEOMAR Helmholtz Center for Ocean Research Kiel, Wischhofstraße 1-3, D-24148 Kiel, Germany

[5]Center for Marine Sensors, Institute for Chemistry and Biology of the Marine Environment (ICBM), Carl von Ossietzky University of Oldenburg, 26382 Wilhelmshaven, Germany

*corresponding author: bsb@icbm.de

Content supporting information: 24 pages (cover page included), 3 text sections, 10 figures, 13 tables

Text Section S1. S³ blanks S-9
Text Section S2. Polymer identification, quantification, and calibration with Py-GC/MS ..S-14
Text Section S3. The residence time of PET particles in the SML.....................................S-20

Fig. S1. S³ remote-controlled research catamaran...S-6
Fig. S2. GPS Data of the S³ in the three sampling areas for each sampling day S-7
Fig. S3. Overview of the materials for various fields of applications, their respective polymer backbones, and identifier ions yielding the various polymer clusters used for polymer quantification...S-11
Fig. S4. Borosilicate substrates used for air sampling. S-15
Fig. S5. Filter cakes of SML and ULW samples with visible fibers...................................S-16
Fig. S6. Mean polymer concentration in µg L^{-1} of the "triplets" of SML and ULW samples with error bars. S-18
Fig. S7. a) One-way ANOVA for SML water samples; b) Multiple comparison test after Tukey for SML water samples. S-19
Fig. S8. a) One-way ANOVA for ULW water samples; b) Multiple comparison test after Tukey for ULW water samples. S-19
Fig. S9. a) pH profile of seawater measured with Unisense microelectrode. b) Experimental set-up of the tanks S-20
Fig. S10. Residence time of small PET particles (< 100 µm) in artificial and real seawater in the SML based on lab experiments. S-21

Table S1. Summary of marine atmospheric MP data, acquired by active sampling published according to Allen et al., 2022, Goßmann et al., 2023, and Caracci et al., 2023......S-4
Table S2. Literature overview of MP documentation in the SML. S-5
Table S3. Overview of air samples. S-7
Table S4. Overview of SML and ULW samples. S-8
Table S5. Plastic standards used for quantification..S-9
Table S6. Conditions for Py-GC/MS. S-10

Table S7. Table of characteristic Py-GC/MS decomposition products used for identification and quantification..S-12
Table S8. Limit of detection (LOD) and limit of quantification (LOQ) for respective polymers analysed in this study.. ....S-13
Table S9. Overview of calibration measurements..S-14
Table S10. Quantitative results of S³ blank in µg L^{-1}..S-15
Table S11. Quantitative results of air samples in ng m^{-3}. S-16
Table S12. Quantitative results of water samples (SML & ULW) in µg L^{-1}. S-17
Table S13. Polymer-specific enrichment factors (EF). S-20

1 Literature overview

Table S1. Summary of marine atmospheric MP data, acquired by active sampling published according to Allen et al., 2022, Goßmann et al., 2023, and Caracci et al., 2023

Reference	Location	MP count [particles m^{-3}]	Size range	Type of analysis	Polymer composition
**Particle-number based studies**					
Ding et al., 2021	South China Sea	0.013 – 0.063	50 µm - 2.21 mm	visual, FTIR	Polyester (29%), Rayon (19%), PP (15%), PE (13%), PS (10%)
Ding et al., 2022	Northwestern Pacific Ocean	0.0046 – 0.064	10 – 4556 µm	µ-FTIR	Rayon (67%), PET (23%)
Ferrero et al., 2022	Baltic Sea and Gotland Island	0 – 85 (301*)	? µm – 5 mm	visual, µ-Raman, FTIR	Polyester (40%), PC (36%), PE (12%), PET (5%), PU (5%)
Liu et al., 2019	Western Pacific Ocean (Shanghai – Mariana Islands)	0 – 1.37	20 µm – 2 mm	visual, FTIR	PET (56%), epoxy resin (10%), PE-PP (7%), PS (6%)
Trainic et al., 2020	North Atlantic Ocean	0 – 0.079	5 µm – 5 mm	µ-Raman and visual (confocal microscope)	PS > PE & PP
Wang et al., 2020	Pearl River Estuary, South China Sea, Indian Ocean	0 – 0.077	58 – 2252 µm	visual, FTIR	PET (50%), PP (22%), other (e.g. phenoxy resin, poly(acrylonitrile-co-acrylic acid), poly(ethylene-co propylene) (28%)
Wang et al., 2021	South China Sea	0 – 0.013	19 – 948 µm	visual, FTIR	PET (55%), PMMA (14%), EVA (9%), PE (9%)
**Reference**	**Location**	**MP mass load [ng m^{-3}]**	**Size range**	**Type of analysis**	**Polymer composition**
**Mass-based studies**					
Caracci et al., 2023	Atlantic Ocean	< LOQ – 51.7	PM_{10}	LC(SEC)-HRMS	PI > PE > PP > PS
Goßmann et al., 2023	Northern Atlantic Ocean	< LOQ – 37.5	> 5 µm	Py-GC/MS	TWP > C-PET$^{\Delta}$ > C-PP$^{\Delta}$ > C-MDI-PUR > C-PS$^{\Delta}$

*max. value detected in the study, with origin from Gdanks Harbour, Poland;

LOQ = limit of quantification; $^{\Delta}$ = partially impaired by secondary contamination

Table S2. Literature overview of MP documentation in the SML.

Author	Sampling Area	Sampling Method	Type of analysis	Result	Accumulation in SML
Ng & Obbard, 2006	Coastal areas, Singapore	Rotating glass drum sampler	FTIR spectroscopy	PE and PS	Not stated
Song et al., 2014	Geoje Island, South Korea	Metal sieve	FTIR spectroscopy	Alkyds (81%), Poly(acrylate/styrene) (11%), PP (2%), Polyester (0.9%), PE (0.8%), Synthetic rubber (0.6%)	Not stated
Song et al., 2015	Jinhae Bay, South Korea	Metal sieve	FTIR spectroscopy	Alkyds (35%), Poly(acrylate/styrene) (16%), PP (8.2%), PE (8.2%), Phenoxy resin (5.6%), PS (5.2%), Polyester (4.7%), Synthetic rubber (0.3%)	Not stated
Anderson et al., 2018	Hamble Estuary and Beaulieu Estuary, UK	Glass plate method & metal sieve	Standard light microscopy, SEM on selected samples	MP fibers only; enrichment in the SML compared to ULW in all sampling locations	yes
Gray et al., 2018	Charleston Harbor and Winyah Bay, South Carolina, USA	Stainless-steel sieve	Dissecting microscope, SEM	Charleston Harbor:<br>413.8 MP particles/m² sediment and 6.6 MP particles/L SML (56% fiber, 26% fragments)<br>Winyah Bay: 221.0 particles/m² sediment and 30.8 particles/L SML (63% fragments, 33% foam)	Not comparable
Stead et al., 2020	Southampton Water, UK	Glass plate method	Optical microscope, FTIR spectroscopy on selected samples	5.9 fibers/m² (62% PE, 4.8% polyvinyl alcohol)	Not stated

FTIR = Fourier-transform infrared spectroscopy; SEM = Scanning electron microscope

2 Sampling and sample information

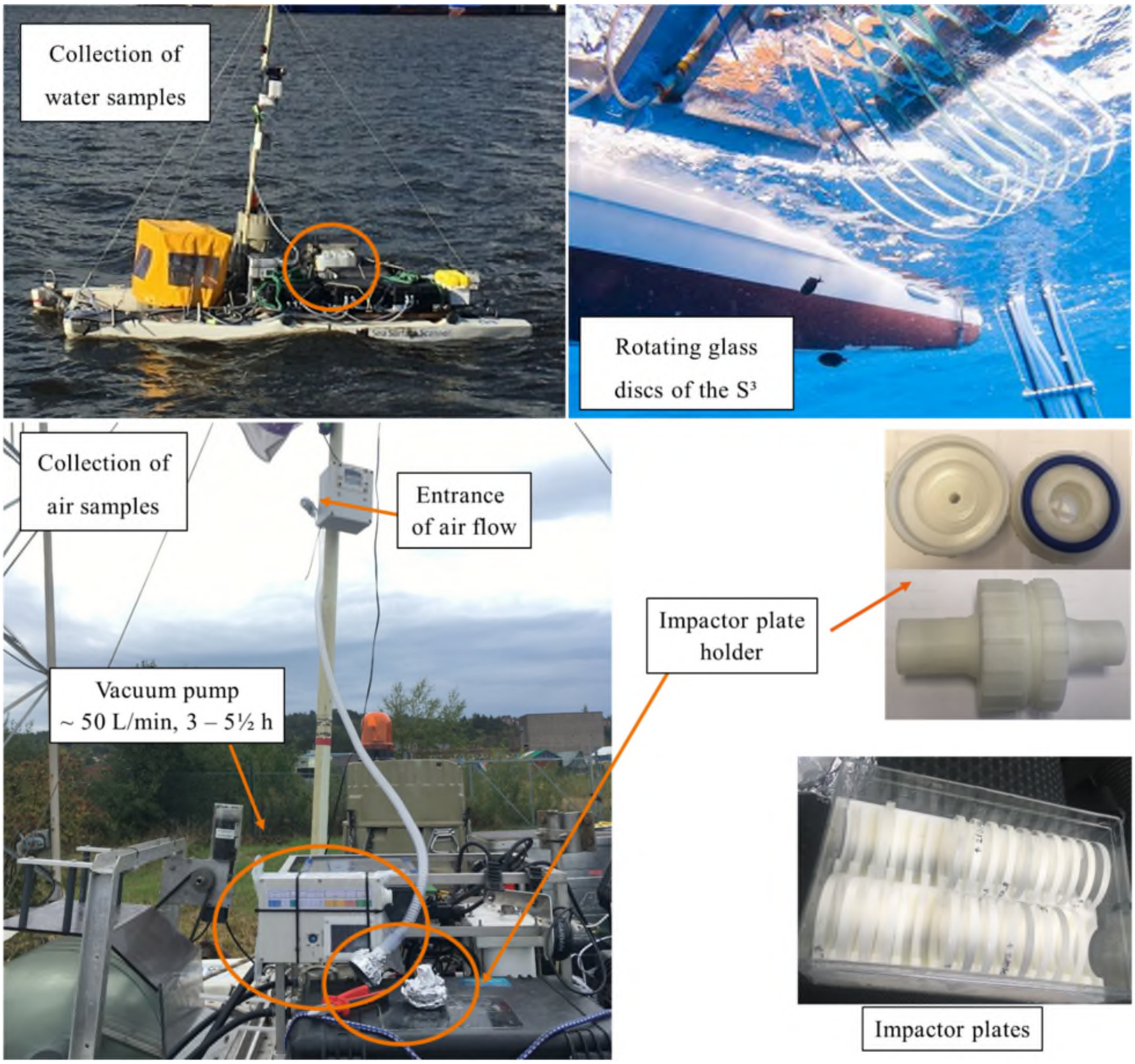

Fig. S1. S³ remote-controlled research catamaran. All pictures were taken by the authors, except the picture on the top right (source: https://uol.de/icbm/prozesse-und-sensorik-mariner-grenzflaechen/equipment-and-infrastructure/sea-surface-scanner-s3, 2020)

Table S3. Overview of air samples including field blanks.

Substrate plate	Sampling Date + Location	Sample type	Total sampling time [h]	Mean volume flow [L min^{-1}]	Total volume [m^3]
**210706_1**	27.09.21, Uddevalla Byfjord, Fjord #1	Blank	00:01	49.06	/
**210706_2**		Sample	04:25	53.55	14.19
**210706_3**	28.09.21, Uddevalla Byfjord, Fjord #1	Blank	00:01	52.71	/
**210706_4**		Sample	05:21	56.73	18.21
**210706_5**	29.09.21, Askerö-fjorden, Fjord #2	Blank	00:01	55.17	/
**210706_6**		Sample	05:31	55.12	18.24
**210706_8**	30.09.21, Askerö-fjorden, Fjord #2	Blank	00:01	53.77	/
**210706_9**		Sample	03:24	53.32	10.88
**210706_10**	01.10.21; Gullmar Fjord, Fjord #3	Blank	00:01	52.77	/
**210706_11**		Sample	03:06	52.79	9.81
**210706_12**	02.10.21; Gullmar Fjord, Fjord #3	Sample	04:20	49.78	12.94
**210706_13***		Blank	00:01	53.14	/

*not measured, fell down in the laboratory

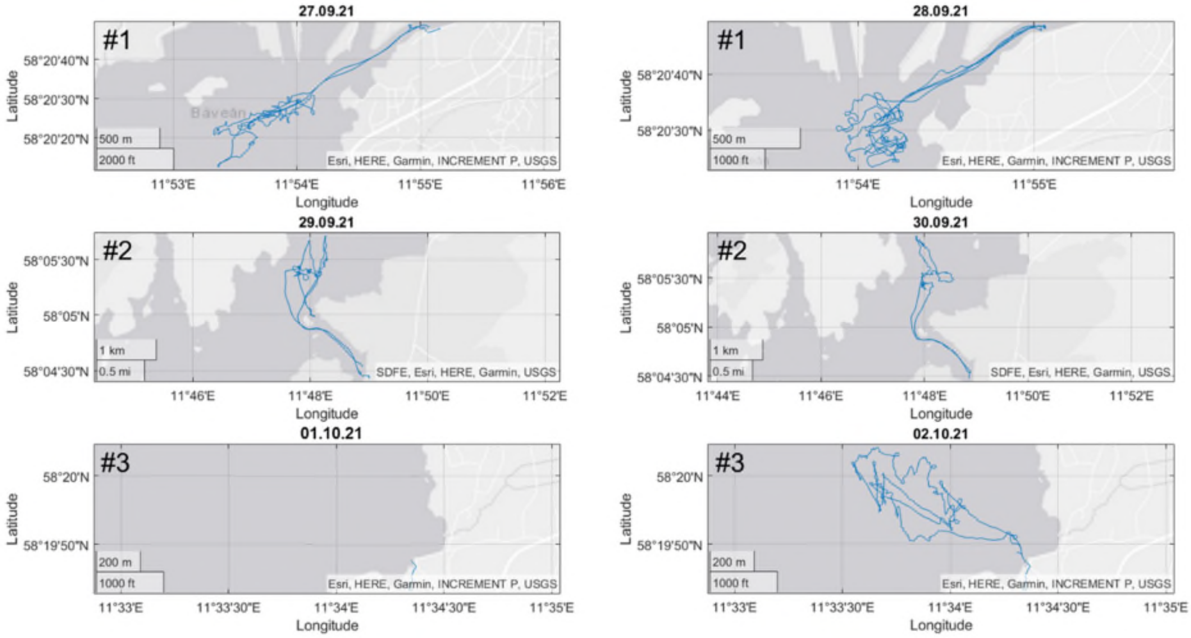

Fig. S2. GPS Data of the S³ in the three sampling areas for each sampling day (#1 Uddevalla Byfjord, #2 Askeröfjorden, #3 Gullmar fjord). The blue trajectories represent the sampling route for the respective days.

Table S4. Overview of SML and ULW samples.

Sampling date + location	Canister Label	Volume [L]	Sampling date + location	Canister Label	Volume [L]
**27.09.21; Uddevalla Byfjord, Fjord #1**	#1 (SML)	9.5	**30.09.21; Askeröfjorden, Fjord #2**	#20 (SML)	8.0
	#2 (SML)	8.5		#21 (SML)	9.1
	#3 (SML)	7.5		#22 (ULW)	7.7
	#4 (ULW)	10.7		#23 (ULW)	10.1
	#5 (ULW)	8.3		#24 (ULW)	8.7
	#6 (ULW)	9.2	**01.10.21; Gullmar Fjord, Fjord #3**	#25 (SML)	8.5
**28.09.21; Uddevalla Byfjord, Fjord #1**	#7 (SML)	8.9		#26 (SML)	8.3
	#8 (SML)	8.0		#27 (SML)	9.0
	#9 (SML)	8.5		#28 (ULW)	6.4
	#10 (ULW)	10.4		#29 (ULW)	8.9
	#11 (ULW)	8.3		#30 (ULW)	8.6
	#12 (ULW)	8.6	**02.10.21; Gullmar Fjord, Fjord #3**	#31 (SML)	8.9
**29.09.21; Askeröfjorden, Fjord #2**	#13 (SML)	7.0		#32 (SML)	8.1
	#14 (SML)	6.0		#33 (SML)	8.3
	#15 (SML)	7.1		#34 (ULW)	8.9
	#16 (ULW)	7.8		#35 (ULW)	9.8
	#17 (ULW)	7.1		#36 (ULW)	9.5
	#18 (ULW)	8.7		#37 (SML)	8.3
**30.09.21; Askeröfjorden, Fjord #2**	#19 (SML)	7.7		#38 (SML)	9.3

Text Section S1. S^3 blanks

Blanks of the S^3 were taken by pumping pre-filtrated water (0.3 µm glass fiber filter, Whatman™; pre-treated in a muffle furnace at 500 °C for 4 h) through the flow-through system of the S^3. for ULW, representing potential contamination for ULW samples (SI, Table S10). Determination of blanks of the SML sampling system could not be performed as blanks were taken on land and glass discs were exposed to elevated levels of dust, resulting in a significant overestimation of the SML blanks. However, SML samples passed through an identical tube and pumping system as ULW with the only difference being the rotating glass discs with PC wipers. As four of the 18 SML samples did not contain any C-PC, a general C-PC contamination originating from the wipers seemed to be unlikely. However, further discussions concerning C-PC require appropriate caution.

3 Methods and measurement details

Table S5. Plastic standards used for quantification.

**Polymer standard**	**Acronym**	**Additional information**	**Supplier**
Polyamide 6 (K891), Alkulon® K222-D	PA6	Low viscosity	Ter Hell, GmbH, Hamburg, Germany
Polycarbonate, Markoplon 2558	PC		Bayer Material, Science
Polyethylene, Lupolen 4261 AG UV	HDPE	High density	LyondellBasell
Polyethylene terephthalate, NEOPET 80	PET		Neogroup
Polymethyl methacrylate, PLEXIGLAS® 7N	PMMA		Plexiglas®
Polypropylene, HL508FB	PP		Borealis
Polystyrene, TOTAL PS impact 7240	PS	High-impact PS for extrusion industry	Ter Hell, GmbH, Hamburg, Germany
Polystyrene, Styrolution PS 158N/L	PS	Raw material	IINEOS Styrosolution
Deuterated poly(styrene-d8) P41212-dPS	dPS		Polymer Source ™, Inc. Canada
Polyurethane	PUR	MDI-PUR	GEBA GmbH
Polyvinylchloride, Vinnolit S3268	PVC	Hard PVC, raw material	Vinnolit
All-season truck tire tread	TTT		Goodyear Tire & Rubber Company
All-season car tire tread	CTT		Semperit (Continental AG)

Table S6. Conditions for Py-GC/MS.

**Micro furnace pyrolyzer & autosampler (EGA/PY-3030D, AS-1020E (FrontierLabs))**	
Carrier gas	Helium
Temperature	590°C
Pyrolysis time	1 min
Transfer line temperature	320°C
**Gas chromatograph (7890B (Agilent))**	
Injector	Split/split less
Mode	Split 1:12.5
Temperature	300°C
Pre-column	Trajan P/N 064062; 10 m x 250 µm/363 µm VSDP tubing
Column	DB5 (J&W); 30 m x 0.25 mm ID, film thickness 0.25 µm
Flow (const.)	1.2 mL min^{-1}
Temperature program	35°C (2 min) → 310°C (30 min) at 4°C min^{-1} → hold 60 min
Transfer line temperature	280°C
**Mass spectrometer (MSD 5977A (Agilent)**	
Ionization energy	70 eV
Scan rate	2.48 scans s^{-1}
Scan range	50-650 amu
EI-Source temperature	230°C
Quadrupole temperature	150°C

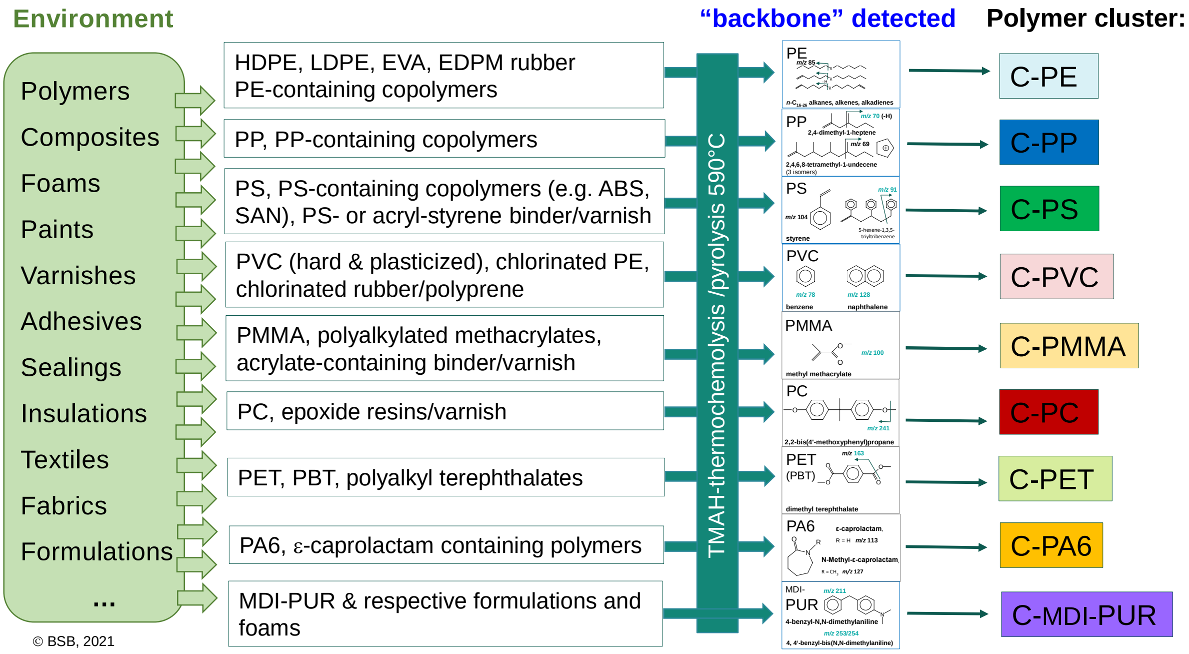

Fig. S3. Overview of the materials for various fields of applications, their respective polymer backbones, and identifier ions yielding the various polymer clusters used for polymer quantification (Primpke et al., 2022). More details are given also in https://analyticalscience.wiley.com/do/10.1002/was.000600517 (Scholz-Böttcher, 2023).

Table S7. Table of characteristic Py-GC/MS decomposition products used for identification and quantification of detected polymer clusters indicated by "C-" as pure polymers (cf. Fig. S3) and potentially included polymer-related derivatives according summarized by Goßmann et al., 2022.

**Basic polymer cluster**	**Cluster associated compounds**	**Characteristic decomposition products**	**Indicator ions [m/z]**
**C-PE**	HDPE, LDPE, PE-containing copolymers and rubbers, ethylene-vinyl acetate (EVA), EPDM rubber	Alkanes (e.g. C_{20})	282 [M], 85
		α-Alkenes (e.g. C_{20})	280 [M], 83
		**α,ω-Alkenes**[a] (e.g. C_{20})	278 [M], 95, **82**
**C-PP**	PP, EPDM rubber	**2,4-Dimethylhept-1-ene**	126 [M], **70**
		2,4,6,8-Tetramethyl-1-undecene[b]	210 [M], 100, 69
		2,4,6,8-Tetramethyl-1-undecene[c]	210 [M], 100, 69
		2,4,6,8-Tetramethyl-1-undecene[d]	210 [M], 100, 69
**C-PS**	PS, PS-containing copolymers (e.g., ABS, SAN), PS- or acryl styrene binders, varnish	Styrene	104 [M]
		2,4-Diphenyl-1-butene	208 [M], 91
		**2,4,6-Triphenyl-1-hexene**	312 [M], **91**
**C-PVC**	PVC (hard and plasticized), chlorinated rubber, chlorinated PE	Benzene	78 [M]
		Chlorobenzene	112 [M]
		**Naphthalene**	**128** [M]
**C-PET**	PET, polybutylene terephthalate (PBT)	**Dimethyl terephthalate**[e]	194 [M], **163**
**C-PMMA**	PMMA; polyalkylated methacrylate, acryl-containing binder	Methacrylate	86 [M], 55
		**Methyl methacrylate**	**100** [M], 69
**C-PC**	PC, epoxide resin	*p*-Methoxy-*tert*-butylbenzene[e]	242 [M], 164, 149
		**2,2-Bis(4'-methoxy-phenyl)propane**[e]	256 [M], **241**
**C-PA6**	PA6	**ε-Caprolactam**	**113** [M]
		**N-methyl caprolactam**[e]	**127** [M]
**C-MDI-PUR**	MDI-PURs, MDI-PUR-based formulations	4,4'-Methylenbis(N-methylaniline)[e]	226 [M]
		N,N-Dimethyl-4-(4-methylamino)benzylanilin[e]	240 [M]
		**4,4'-Methylenbis(N,N-dimethylaniline)**[e]	**254** [M], 253, 210, 134
**TTT**	Truck tire tread, bus tire tread	**2,4-Dimethyl-4-vinylcyclohexene (DMVCH)**	136 [M], 121, 93, **68**
		1-Methyl-4-(1-methylethenyl)-cyclohexene	**136** [M], 121, 93, 68
**CTT**	Car tire tread	Ethenylbenzene	104 [M], 78, 51
		**Cyclohexenylbenzene (SB)**	158 [M], 129, 115, **104**
**dPS**	Deuterated polystyrene used as internal standard for pyrolysis	Styrene (deuterated)	112 [M]
		2,4-Diphenyl-1-butene (deuterated)	224 [M], 98
		**2,4,6-Triphenyl-**1-hexene (deuterated)	336 [M], **98**

[m/z] = mass to charge ratio; [M] = molecular ion; bold = indicator ions used for calibration and quantification; [a]Mean of *n*-C_{16}-C_{26}-alkadiens used for quantification of PE. [b]isotactic. [c]heterotactic. [d]syndiotactic. [e]only after TMAH treatment.

Table S8. Limit of detection (LOD) and limit of quantification (LOQ) for respective polymers analysed in this study. S/N calculation based on the peak heights of the respective indicator ions for each basis polymer given in Table S7. The data are representative for a system in optimum condition.

**Polymer**	**Limit of detection (LOD) S/N =3**<br>**(ng absolute)**	**Limit of quantification (LOQ) S/N =10**<br>**(ng absolute)**
**C-PE**[c]	100-300	100-300
**C-PP**[c]	< 100[b]	500
**C-PET**[a]	10-50	50
**C-PS**[a]	1	2
**C-PVC**[a]	10-50	50
**C-PMMA**	< 100[b]	100-300
**C-PC**[a]	1	2
**C-MDI-PUR**[c]	100-300[b]	500
**C-PA6**[c]	100-300[b]	500
**CTT**[c]	< 5000	20 000
**TTT**[c]	< 1000[c]	< 1000[c]

[a]based on dissolved standards; [b]derived from lowest calibration point for orientation only; [c]lower calibration impossible due to particulate standard. With respect to CTT and TTT that are both used as entire tire treads see also Goßmann et al. 2021.

Text Section S2. Polymer identification, quantification, and calibration with Py-GC/MS

Particulate standards were weighed in with a Cubis ultramicro balance MSE2.7S-000-DM (Sartorius, Germany; readability 0.0001 mg, repeatability 0.00025 mg). The calibration range was between 1 and 50 µg. For PS, PC, PET, and PVC dissolved standards in concentrations down to 0.05 µg were applied additionally. CTT formed an exception with a calibration range from 20 to 200 µg due to its relatively high limit of detection (LOD, 20 µg, since applied as entire tire tread, cf. (Goßmann et al., 2021)) for the characteristic decomposition product combined with high-expected mass loads. To all measured standards, 2 µg dPS (absolute) as internal standard, and 20 µL TMAH 12.5% in methanol for thermochemolysis were added. Internal standardized calibration curves were created for each polymer individually; here, for each concentration corresponding peak ratios were calculated based on the respective peak area of the corresponding polymer indicator signal divided by the peak area of the dPS trimer indicator signal (*m/z* 98). For further details, refer to Fischer & Scholz-Böttcher, 2019; Goßmann et al., 2021.

Table S9. Overview of calibration measurements.

	**PE**	**PP**	**PET**	**PS**	**PVC**	**PMMA**	**PA6**	**PC**	**MDI-PUR**	**CTT**	**TTT**
Date of measurement sequence: **17.11.21**											
**B**	-0,0173	-0,0673	0,0888	0,0755	0,2751	0,4289		-0,1684	0,0057	-0,0663	-0,0619
**Slope**	0,0128	0,6322	1,6327	0,6675	0,0430	0,3250		5,8832	0,0373	0,0039	0,0523
**r²**	0,9598	0,6588	0,6718	0,9246	0,5184	0,6400		0,9021	0,3447	0,9281	0,8817
Date of measurement sequence: **18.02.22**											
**B**	-0,0049	-0,0673	0,0888	0,0755	0,1729	0,4311	-0,4926	-2,0356	0,0057	-0,0663	-0,0619
**Slope**	0,0177	0,6322	1,6327	0,6675	0,0271	0,4572	0,4892	12,0714	0,0373	0,0039	0,0523
**r²**	0,4938	0,6588	0,6718	0,9246	0,4699	0,8687	0,9243	0,9904	0,3447	0,9281	0,8817
Date of measurement sequence: **01.03.22**											
**B**	-0,0259	-0,0673	0,0888	0,0755	0,0874	0,8855		-0,9442	0,0057	-0,0663	-0,0619
**Slope**	0,0274	0,6322	1,6327	0,6675	0,0480	0,4373		20,8900	0,0373	0,0039	0,0523
**r²**	0,7759	0,6588	0,6718	0,9246	0,9374	0,8473		0,9862	0,3447	0,9281	0,8817
Date of measurement sequence: **28.03.22**											
**B**	-0,0049	-0,0673	0,0888	0,0755	0,0086	0,4584		-1,8099	0,0057	-0,0663	-0,0619
**Slope**	0,0177	0,6322	1,6327	0,6675	0,0362	0,1169		16,9411	0,0373	0,0039	0,0523
**r²**	0,4938	0,6588	0,6718	0,9246	0,7912	0,8736		0,8872	0,3447	0,9281	0,8817
Date of measurement sequence: **25.05.22**											
**B**	-0,0081	-0,0673	0,0888	0,0755	0,1465	0,3322		-0,1388	0,0528	-0,0663	-0,0619
**Slope**	0,0167	0,6322	1,6327	0,6675	0,0414	0,1477		7,7390	0,0229	0,0039	0,0523
**r²**	0,7775	0,6588	0,6718	0,9246	0,8556	0,9192		0,9717	0,6419	0,9281	0,8817

B = y-intercept, r² = coefficient of determination

4 Detailed sample results

Table S10. Quantitative results of S³ blank in µg L^{-1}.

S³ Blanks	C-PE	C-PP	C-PET	C-PS	C-PVC	C-PC	C-PMMA	C-PA6	C-MDI-PUR	CTT	TTT
	µg L^{-1}										
#1	n.d.	n.d.	0.01	n.q.	n.q.	< 0.01	1.27	n.d.	n.d.	n.d.	n.d.
#2	n.d.	n.d.	n.q.	n.q.	n.q.	< 0.01	3.57	n.d.	n.d.	n.d.	n.d.
#3	n.d.	n.d.	n.d.	n.q.	n.q.	n.q.	0.51	n.d.	n.d.	n.d.	n.d.
#4	n.d.	n.d.	0.01	n.q.	n.q.	n.q.	0.51	n.d.	n.d.	n.d.	n.d.
#5	n.d.	n.d.	n.q.	n.q.	n.q.	n.q.	n.q.	n.d.	n.d.	n.d.	n.d.

n.d. = not detectable, n.q. = not quantifiable; Quantification was based on calibration curves from Table S9 (25.05.22). The measurement sequence of the S³ blanks did not allow a reliable quantification.

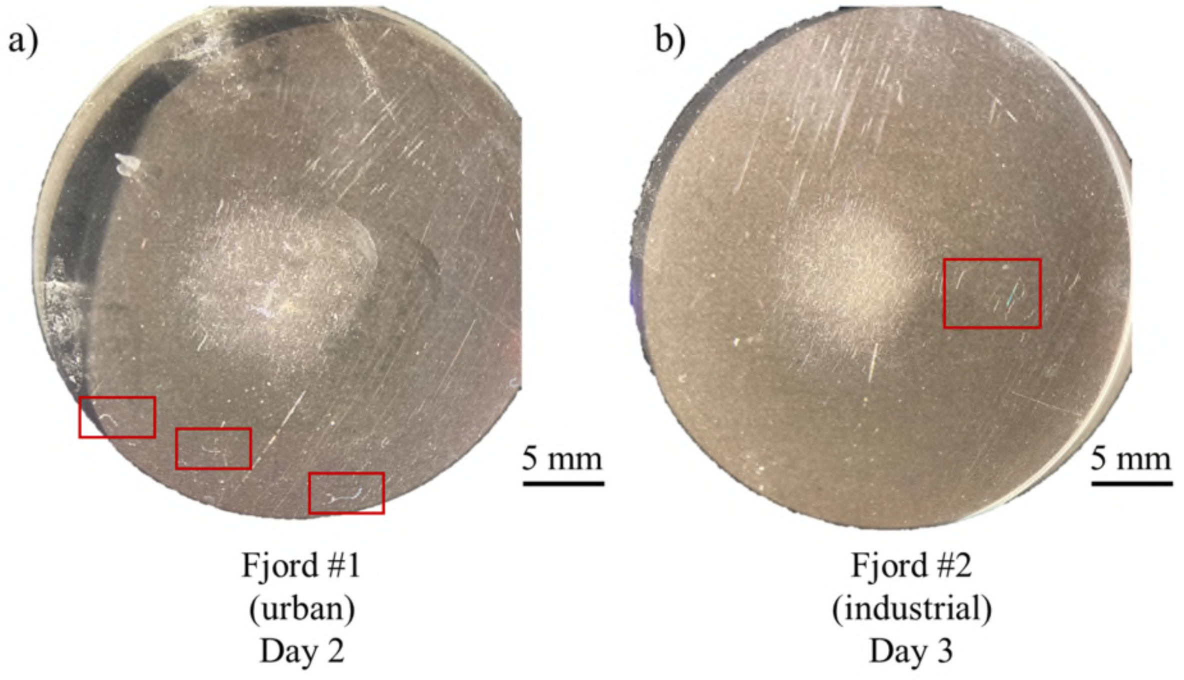

Fig. S4. Borosilicate substrates used for air sampling (ø 30 mm) with visible fibers. a) Sample from fjord #1 (urban), Day 2, and b) sample from fjord #2 (industrial), Day 3.

Table S11. Quantitative results of air samples in ng m^{-3}. C-PE, C-PA6, C-MDI-PUR, CTT, and TTT are not included as they were not detectable in the air.

Sample	C-PP	C-PET	C-PS	C-PVC	C-PC	C-PMMA
	ng m^{-3}					
**Fjord #1 Day 1**	n.d.	25.71	1.15	298.12	3.13	18.74
**Fjord #1 Day 2**	5.05	8.08	0.04	51.87	1.11	n.q.
**Fjord #2 Day 3**	n.q.	5.60	n.q.	43.72	1.14	n.q.
**Fjord #2 Day 4**	n.d.	10.45	0.28	125.92	1.87	n.q.
**Fjord #3 Day 5**	n.d.	n.q.	n.q.	n.q.	2.06	n.q.
**Fjord #3 Day 6**	n.d.	n.q.	0.24	n.q.	n.q.	n.q.

n.d. = not detectable, n.q. = not quantifiable

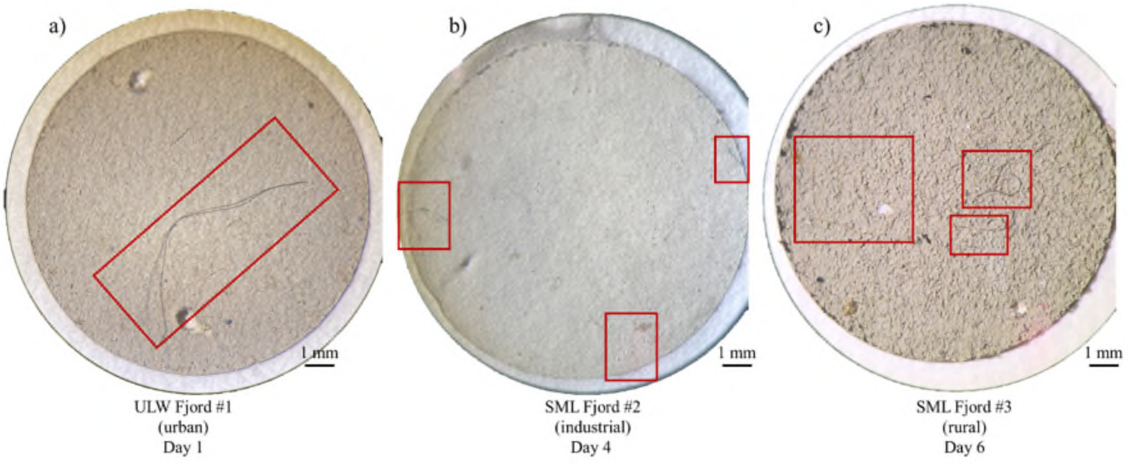

Fig. S5. Filter cakes of SML and ULW samples with visible fibers. a) ULW sample from Fjord #1 (urban), Day 1, and b) SML sample from Fjord #2 (industrial), Day 4, and c) SML sample from Fjord #3 (rural), Day 6.

Table S12. Quantitative results of water samples (SML & ULW) in µg L^{-1}.

Sample	C-PE	C-PP	C-PET	C-PS	C-PVC	C-PC	C-PMMA	C-PA6	C-MDI-PUR	CTT	TTT
**SML**	µg L^{-1}										
**Fjord #1 Day 1**	0.41	0.11	1.44	0.05	5.25	0.01	1.27	n.d.	n.q.	6.87	0.17
**Fjord #1 Day 2**	0.19	0.10	0.66	0.01	6.19	n.q.	3.57	n.d.	n.d.	2.16	0.05
**Fjord #2 Day 3**	0.32	0.05	0.15	n.q.	4.94	n.q.	0.51	n.d.	n.d.	3.60	0.16
**Fjord #2 Day 4**	0.52	0.11	0.19	n.q.	5.34	n.q.	0.51	n.d.	0.04	7.04	n.q.
**Fjord #3 Day 5**	0.05	0.10	0.14	n.q.	1.55	0.01	n.q.	n.d.	n.d.	0.74	n.d.
**Fjord #3 Day 6**	0.17	0.08	1.10	n.q.	2.80	0.02	0.09	0.11	n.q.	1.31	n.d.
**ULW**	µg L^{-1}										
**Fjord #1 Day 1**	0.14	0.09	0.55	0.02	1.39	0.01	0.36	n.d.	n.d.	0.72	0.05
**Fjord #1 Day 2**	0.12	0.02	0.84	0.01	4.30	0.01	8.11	n.d.	n.d.	0.85	n.d.
**Fjord #2 Day 3**	0.13	0.01	0.31	n.q.	1.87	0.01	9.21	n.d.	n.d.	1.11	n.d.
**Fjord #2 Day 4**	0.23	0.04	0.23	n.q.	4.67	0.01	0.23	0.27	0.18	2.40	0.17
**Fjord #3 Day 5**	0.10	0.06	0.03	n.q.	4.72	n.q.	0.38	n.d.	0.02	n.d.	n.d.
**Fjord #3 Day 6**	0.16	0.06	0.42	0.03	2.81	0.01	2.05	0.09	n.d.	0.56	n.d.

n.d. = not detectable, n.q. = not quantifiable

5 General considerations

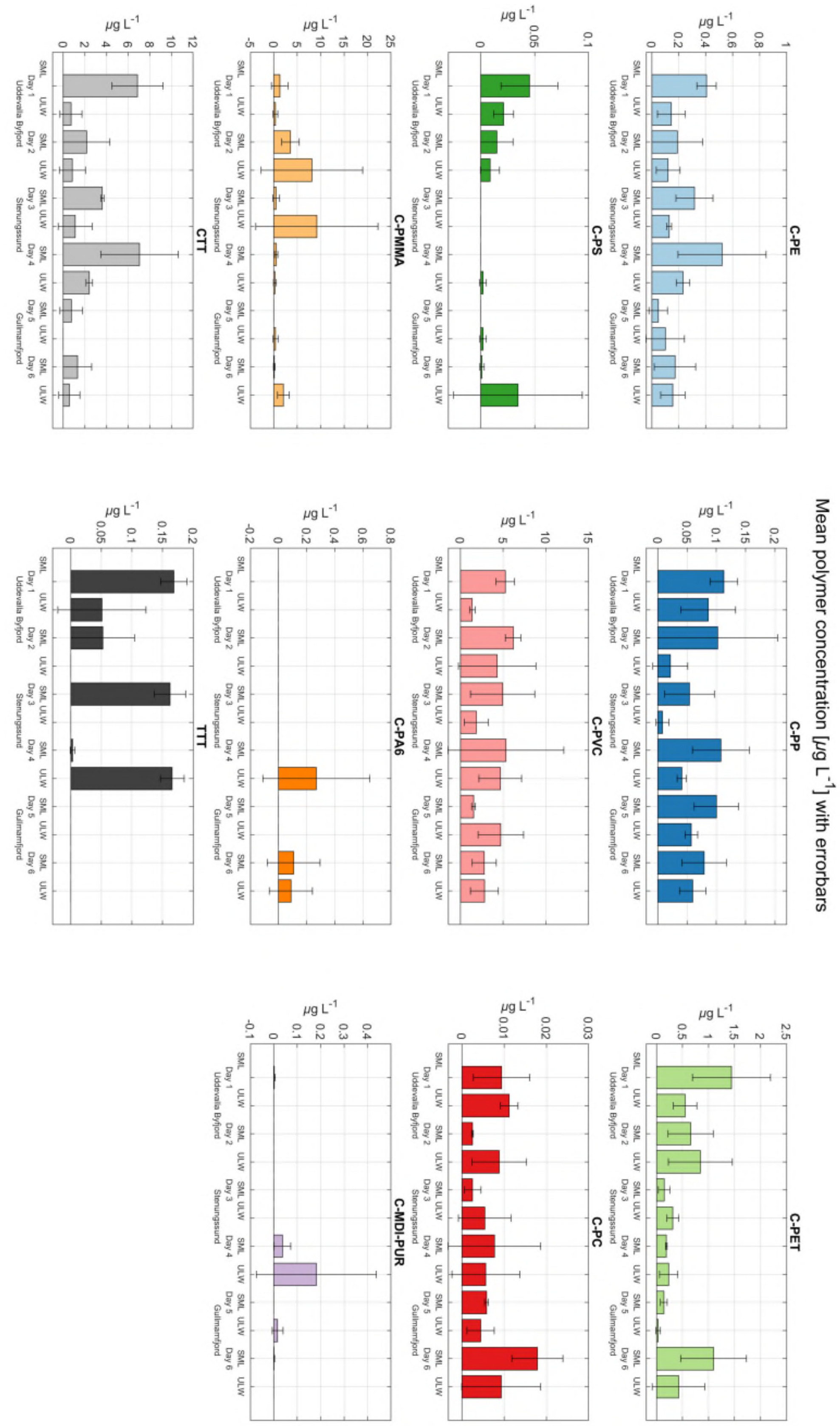

Fig. S6. Mean polymer concentration in µg L^{-1} of the “triplets” of SML and ULW samples with error bars.

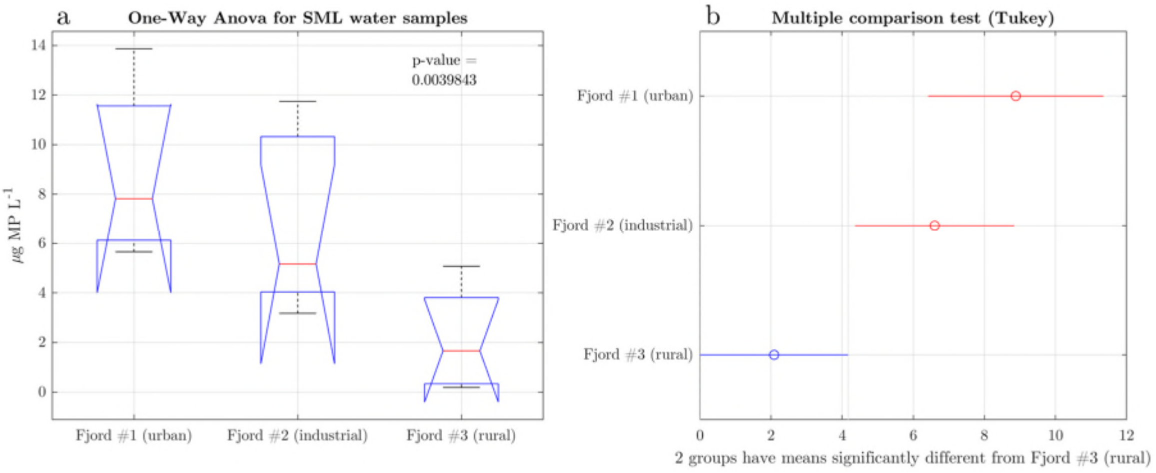

Fig. S7. a) One-way ANOVA for SML water samples; b) Multiple comparison test after Tukey for SML water samples.

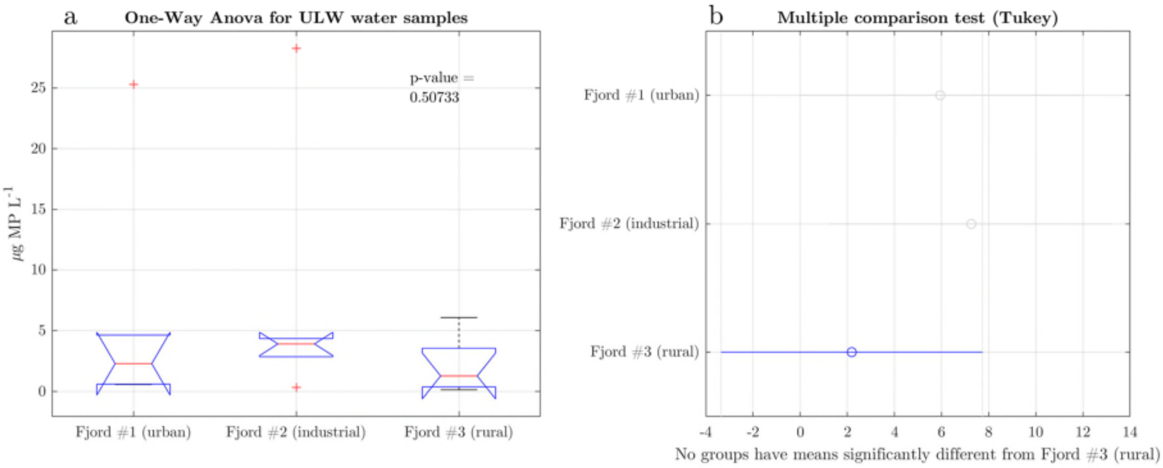

Fig. S8. a) One-way ANOVA for ULW water samples; b) Multiple comparison test after Tukey for ULW water samples.

Table S13. Polymer-specific enrichment factors (EF).

	C-PE	C-PP	C-PET	C-PS	C-PVC	C-PC	C-PMMA	CTT	TTT	TWP
**Fjord #1 Day 1**	2.8	1.3	2.6	2.1	3.8	0.8	3.5	9.6	3.3	9.2
**Fjord #1 Day 2**	1.6	4.8	0.8	1.7	1.4	0.3	0.4	2.6	+	2.6
**Fjord #2 Day 3**	2.5	7.0	0.5	/	2.6	0.5	0.1	3.2	+	3.4
**Fjord #2 Day 4**	2.3	2.6	0.8	-	1.1	1.4	2.3	2.9	<0.1	2.7
**Fjord #3 Day 5**	0.5	1.8	5.4	-	0.3	1.3	-	+	/	+
**Fjord #3 Day 6**	1.1	1.3	1.9	< 0.1	1.0	1.9	< 0.1	2.3	/	2.3

+ = only detected in SML; - = only detected in ULW, / = not detected

Text Section S3. The residence time of PET particles in the SML

Lab experiments were conducted to generate knowledge about the residence time of small PET particles below 100 µm in the SML. For the experimental set-up, four 10 L tanks were covered with Teflon foil to avoid the adherence of particles on the glass of the tanks. Then they were thoroughly rinsed with pre-filtered water and ethanol (96%). Each tank was filled with pre-filtered (artificial) seawater (pore size 1 µm) and covered with aluminum foil. In a separate container, the thickness of the SML in the respective seawater was measured with microelectrodes (pH-microelectrode, Unisense A/S, Denmark). The SML is detectable based on its pH value which differs slightly from the underlying water (Zhang et al., 2003). According to the pH profile (Fig. S9 a)), the SML had a thickness below 50 µm. Together with the size of the tank, the total volume of the SML was calculable.

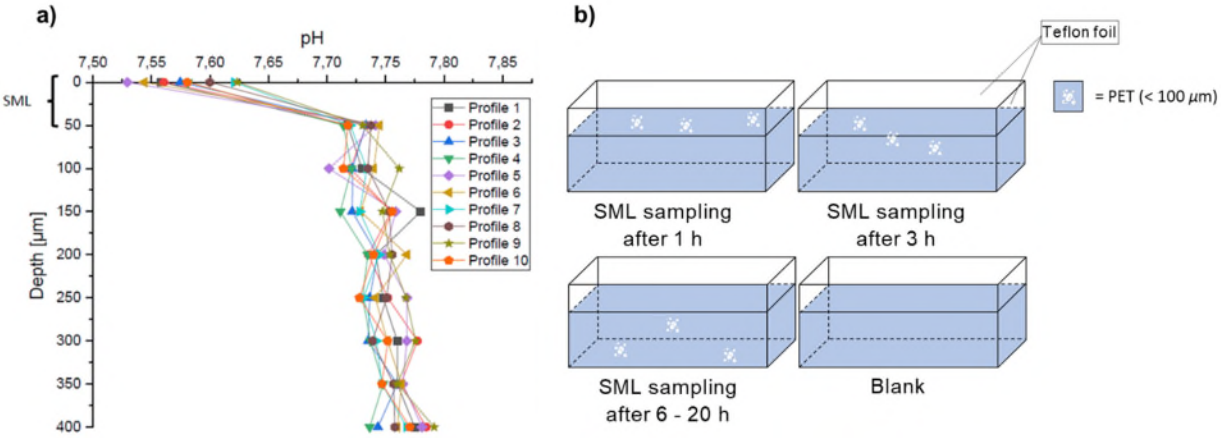

Fig. S9. a) pH profile of seawater measured with Unisense microelectrode. b) Experimental set-up of the tanks

After determining the thickness of the SML, the 50 µg of PET particles were carefully deposited on the surface of three of the four tanks. The fourth one was used as a blank (Fig. S9 b)). After 1 hour, 3 hours, and 6 to 20 hours the entire SML of the respective tanks was sampled, filtrated on glass fiber filters, and measured with Py-GC/MS. Afterwards, PET was quantified as described in the manuscript. The results are displayed in Fig. S10. Experiments were conducted with seawater (n = 6) and artificial seawater (n = 3). In both cases, PET particles quickly left the SML and after 20 hours, almost no PET was left in the SML.

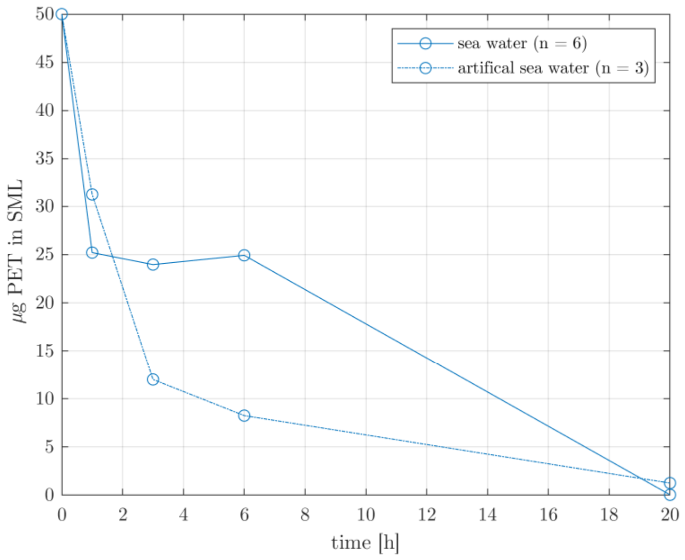

Fig. S10. Residence time of small PET particles (< 100 µm) in artificial and real seawater in the SML based on lab experiments.

6 Additional references of supplemental information

Allen, D., Allen, S., Abbasi, S., Baker, A., Bergmann, M., Brahney, J., Butler, T., Duce, R. A., Eckhardt, S., Evangeliou, N., Jickells, T., Kanakidou, M., Kershaw, P., Laj, P., Levermore, J., Li, D., Liss, P., Liu, K., Mahowald, N., … Wright, S. (2022). Microplastics and nanoplastics in the marine-atmosphere environment. *Nature Reviews Earth & Environment*, *3*(6), 393–405. https://doi.org/10.1038/s43017-022-00292-x

Anderson, Z. T., Cundy, A. B., Croudace, I. W., Warwick, P. E., Celis-Hernandez, O., & Stead, J. L. (2018). A rapid method for assessing the accumulation of microplastics in the sea surface microlayer (SML) of estuarine systems. *Scientific Reports*, *8*(1), 9428. https://doi.org/10.1038/s41598-018-27612-w

Caracci, E., Vega-Herrera, A., Dachs, J., Berrojalbiz, N., Buonanno, G., Abad, E., Llorca, M., Moreno, T., & Farré, M. (2023). Micro(nano)plastics in the atmosphere of the Atlantic Ocean. *Journal of Hazardous Materials*, *450*(February), 131036. https://doi.org/10.1016/j.jhazmat.2023.131036

Ding, J., Sun, C., He, C., Zheng, L., Dai, D., & Li, F. (2022). Atmospheric microplastics in the Northwestern Pacific Ocean: Distribution, source, and deposition. *Science of The Total Environment*, *829*, 154337. https://doi.org/10.1016/j.scitotenv.2022.154337

Ding, Y., Zou, X., Wang, C., Feng, Z., Wang, Y., Fan, Q., & Chen, H. (2021). The abundance and characteristics of atmospheric microplastic deposition in the northwestern South China Sea in the fall. *Atmospheric Environment*, *253*, 118389. https://doi.org/10.1016/j.atmosenv.2021.118389

Ferrero, L., Scibetta, L., Markuszewski, P., Mazurkiewicz, M., Drozdowska, V., Makuch, P., Jutrzenka-Trzebiatowska, P., Zaleska-Medynska, A., Andò, S., Saliu, F., Nilsson, E. D., & Bolzacchini, E. (2022). Airborne and marine microplastics from an oceanographic survey at the Baltic Sea: An emerging role of air-sea interaction? *Science of The Total Environment*, *824*, 153709. https://doi.org/10.1016/j.scitotenv.2022.153709

Fischer, M., & Scholz-Böttcher, B. M. (2019). Microplastics analysis in environmental samples-recent pyrolysis-gas chromatography-mass spectrometry method improvements to increase the reliability of mass-related data. *Anal. Methods*, *11*(18), 2489–2497. https://doi.org/10.1039/c9ay00600a

Goßmann, I., Halbach, M., & Scholz-Böttcher, B. M. (2021). Car and truck tire wear particles in complex environmental samples – A quantitative comparison with “traditional” microplastic polymer mass loads. *Science of The Total Environment*, *773*, 145667. https://doi.org/10.1016/j.scitotenv.2021.145667

Goßmann, I., Herzke, D., Held, A., Schulz, J., Nikiforov, V., Georgi, C., Evangeliou, N., Eckhardt, S., Gerdts, G., Wurl, O., & Scholz-Böttcher, B. M. (2023). Occurrence and backtracking of microplastic mass loads including tire wear particles in northern Atlantic air. *Nature Communications*, *14*(1), 3707. https://doi.org/10.1038/s41467-023-39340-5

Goßmann, I., Süßmuth, R., & Scholz-Böttcher, B. M. (2022). Plastic in the air?! - Spider webs as spatial and temporal mirror for microplastics including tire wear particles in urban air. *Science of The Total Environment*, *832*(January), 155008. https://doi.org/10.1016/j.scitotenv.2022.155008

Gray, A. D., Wertz, H., Leads, R. R., & Weinstein, J. E. (2018). Microplastic in two South Carolina Estuaries: Occurrence, distribution, and composition. *Marine Pollution Bulletin*, *128*(January), 223–233. https://doi.org/10.1016/j.marpolbul.2018.01.030

Liu, K., Wu, T., Wang, X., Song, Z., Zong, C., Wei, N., & Li, D. (2019). Consistent Transport of Terrestrial Microplastics to the Ocean through Atmosphere. *Environmental Science & Technology*, *53*(18), 10612–10619. https://doi.org/10.1021/acs.est.9b03427

Ng, K. L., & Obbard, J. P. (2006). Prevalence of microplastics in Singapore's coastal marine environment. *Marine Pollution Bulletin*, *52*(7), 761–767. https://doi.org/10.1016/j.marpolbul.2005.11.017

Primpke, S., Booth, A. M., Gerdts, G., Gomiero, A., Kögel, T., Lusher, A., Strand, J., Scholz-Böttcher, B. M., Galgani, F., Provencher, J., Aliani, S., Patankar, S., & Vorkamp, K. (2022). Monitoring of microplastic pollution in the Arctic: recent developments in polymer identification, quality assurance and control, and data reporting. *Arctic Science*, *22*(2022), 1–22. https://doi.org/10.1139/as-2022-0006

Scholz-Böttcher, B. M. (2023, April). Mass-based methods for the analysis of micro- and nanoplastics. *Wiley Analytical Science Magazine*. https://doi.org/do/10.1002/was.00050419

Song, Y. K., Hong, S. H., Jang, M., Han, G. M., & Shim, W. J. (2015). Occurrence and Distribution of Microplastics in the Sea Surface Microlayer in Jinhae Bay, South Korea. *Archives of Environmental Contamination and Toxicology*, *69*(3), 279–287. https://doi.org/10.1007/s00244-015-0209-9

Song, Y. K., Hong, S. H., Jang, M., Kang, J.-H., Kwon, O. Y., Han, G. M., & Shim, W. J. (2014). Large Accumulation of Micro-sized Synthetic Polymer Particles in the Sea Surface Microlayer. *Environmental Science & Technology*, *48*(16), 9014–9021. https://doi.org/10.1021/es501757s

Stead, J. L., Cundy, A. B., Hudson, M. D., Thompson, C. E. L., Williams, I. D., Russell, A. E., & Pabortsava, K. (2020). Identification of tidal trapping of microplastics in a temperate salt marsh system using sea surface microlayer sampling. *Scientific Reports*, *10*(1), 14147.

https://doi.org/10.1038/s41598-020-70306-5

Trainic, M., Flores, J. M., Pinkas, I., Pedrotti, M. L., Lombard, F., Bourdin, G., Gorsky, G., Boss, E., Rudich, Y., Vardi, A., & Koren, I. (2020). Airborne microplastic particles detected in the remote marine atmosphere. *Communications Earth & Environment*, *1*(1), 64. https://doi.org/10.1038/s43247-020-00061-y

Wang, X., Li, C., Liu, K., Zhu, L., Song, Z., & Li, D. (2020). Atmospheric microplastic over the South China Sea and East Indian Ocean: abundance, distribution and source. *Journal of Hazardous Materials*, *389*, 121846. https://doi.org/10.1016/j.jhazmat.2019.121846

Wang, X., Liu, K., Zhu, L., Li, C., Song, Z., & Li, D. (2021). Efficient transport of atmospheric microplastics onto the continent via the East Asian summer monsoon. *Journal of Hazardous Materials*, *414*, 125477. https://doi.org/10.1016/j.jhazmat.2021.125477

Zhang, Z., Cai, W., Liu, L., Liu, C., & Chen, F. (2003). Direct determination of thickness of sea surface microlayer using a pH microelectrode at original location. *Sci. China, Ser. B Chem.*, *46*(4), 339–351. https://doi.org/10.1360/02yb0192
